# Controlling alkyne reactivity by means of a copper-catalyzed radical reaction system for the synthesis of functionalized quaternary carbons

**DOI:** 10.3762/bjoc.16.45

**Published:** 2020-03-26

**Authors:** Goki Hirata, Yu Yamane, Naoya Tsubaki, Reina Hara, Takashi Nishikata

**Affiliations:** 1 Graduate School of Science and Engineering, Yamaguchi University 2-16-1 Tokiwadai, Ube, Yamaguchi, 755-8611, Japan

**Keywords:** copper catalyst, 1,3-enyne, functionalized quaternary carbon, indolinone, tandem alkyl radical addition

## Abstract

A terminal alkyne is one of the most useful reactants for the synthesis of alkyne and alkene derivatives. Because an alkyne undergoes addition reaction at a C–C triple bond or cross-coupling at a terminal C–H bond. Combining those reaction patterns could realize a new reaction methodology to synthesize complex molecules including C–C multiple bonds. In this report, we found that the reaction of 3 equivalents of terminal alkyne **1** (aryl substituted alkyne) and an α-bromocarbonyl compound **2** (tertiary alkyl radical precursor) undergoes tandem alkyl radical addition/Sonogashira coupling to produce 1,3-enyne compound **3** possessing a quaternary carbon in the presence of a copper catalyst. Moreover, the reaction of α-bromocarbonyl compound **2** and an alkyne **4** possessing a carboxamide moiety undergoes tandem alkyl radical addition/C–H coupling to produce indolinone derivative **5**.

## Introduction

Terminal alkynes are undoubtedly useful functional groups for organic synthesis, and they can undergo a variety of reactions [1]. The C–C triple bond of an alkyne is suitable for addition reactions, whereas the terminal hydrogen atom is a good target for cross-coupling by using Sonogashira and related coupling reactions [2–4]. Although there are many reports on alkyne transformations, one recent development in this area has been the reaction of alkynes with tertiary alkyl electrophiles to produce functionalized quaternary carbon atoms via addition [5–10] or coupling [11–16].

**Scheme 1:**
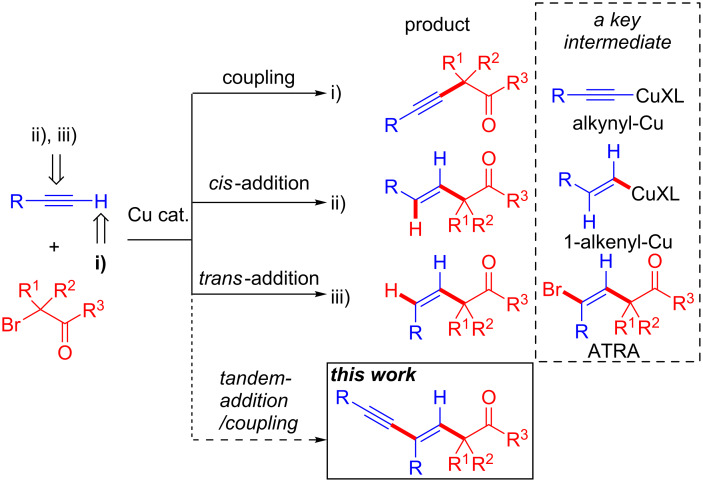
Reaction modes of alkyne.

Recently, we have prepared quaternary carbon centers via radical reactions by using α-bromocarbonyl compounds (a tertiary alkyl source) and olefins or heteroatoms in the presence of a copper catalyst [17–19]. During our studies, we found that combinations of alkynes and tertiary alkyl radicals generated from the reaction of a copper catalyst and an α-bromocarbonyl compound can undergo i) Sonogashira type couplings via an alkynyl-Cu intermediate [20], ii) *cis*-hydro tertiary alkylations via 1-alkenyl-Cu [21], and iii) *trans*-hydro tertiary alkylations via atom-transfer radical addition (ATRA) [21] (Scheme 1, i–iii). Therefore, we postulated that if we could control the reactivities of the alkynyl–Cu and ATRA adducts, a tandem tertiary alkylation followed by an alkynylation could occur to produce a 1,3-enyne possessing a quaternary carbon center with good regio- and stereoselectivity (Scheme 1, this work). Similarly, Zhu’s group has reported that the reaction of an alkyne and an α-bromocarbonyl compound furnishes a highly functionalized 1,3-enyne compound via ATRA followed by an alkynylation reaction [22], but both Pd and Cu are required as catalysts in that case. Our methodology can realize a Pd-free catalyst system to prepare complex quaternary carbon atoms. Herein, we report the Cu-catalyzed control of the reactivity of an alkyne (addition and coupling) undergoing tandem tertiary alkylation and alkynylation to produce a 1,3-enyne containing a quaternary carbon center with good regio- and stereoselectivity.

## Results and Discussion

In our preliminary research, we tried various Cu salts, including CuI, CuBr, CuCl, CuOAc, and CuOTf, and ligands, including PPh_3_, 1,10-phenanthroline (1,10-Phen), *N*,*N*,*N*',*N*'',*N*''-pentamethyldiethylenetriamine, and tris(2-pyridylmethyl)amine, in the reaction of phenylacetylene (**1a**) and α-bromoester **2a**. From these experiments, CuBr and 1,10-Phen acted as a good catalyst system for obtaining 1,3-enyne **3a** in 62% yield with good regio- and stereoselectivity (Table 1, entry 1). On the basis of this preliminary result, we also tried other conditions. Toluene was very effective in our previous reaction system [17] but was not effective at all in this case (Table 1, entry 2). The reaction without NaI resulted in the formation of **3a**-**Br** in 30% yield, instead of **3a** (Table 1, entry 3). If KI was used instead of NaI, the yield of **3a** decreased (Table 1, entry 4). We will discuss the proposed reaction mechanism later in the text, but the formation of **3a**-**I** via ATRA could be important for the alkynylation reaction. Generally, the Sonogashira coupling requires both a Pd catalyst and a Cu co-catalyst [2–4]. However, couplings with terminal alkynes can be carried out in the absence of the Pd catalyst [23–32]; this is the so-called Castro–Stephens reaction [33]. The effect of the base was very important for producing the main product **3a** (Table 1, entries 5–8). If the reaction was performed in the presence of a base other than Cs_2_CO_3_, a decreased yield of **3a** was observed. Finally, an increased amount of catalyst was effective for obtaining the highest yield (Table 1, entry 9). The total yield was moderate, but the yields for each step of this two-step tandem reaction system (ATRA followed by Castro–Stephens coupling) should be over 80%.

**Table 1:** Optimization.^a^

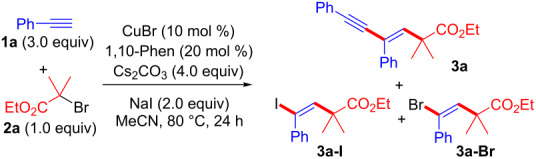				

entry	changes from standard conditions	**3a** (%)	**3a-I** (%)	**3a-Br** (%)

1	none	62	10	trace
2	toluene instead of MeCN	trace	–	–
3	without NaI	<5	0	30
4	KI instead of NaI	30	7	7
5	Hunig’s base instead of Cs_2_CO_3_	trace	60	trace
6	iPr_2_NH instead of Cs_2_CO_3_	trace	65	trace
7	K_2_CO_3_ instead of Cs_2_CO_3_	6	58	trace
8	K_3_PO_4_ instead of Cs_2_CO_3_	26	20	trace
9	15 mol % CuBr and 30 mol % 1,10-Phen	66(52)^b^	7	trace

^a^Conducted at 80 °C for 24 h in MeCN with CuBr (10 mol %), 1,10-Phen (20 mol %), Cs_2_CO_3_ (4.0 equiv), NaI (2.0 equiv), **1a** (3.0 equiv) and **2a** (1.0 equiv). Yields were determined by ^1^H NMR analysis. ^b^GPC yield.

**Figure 1:**
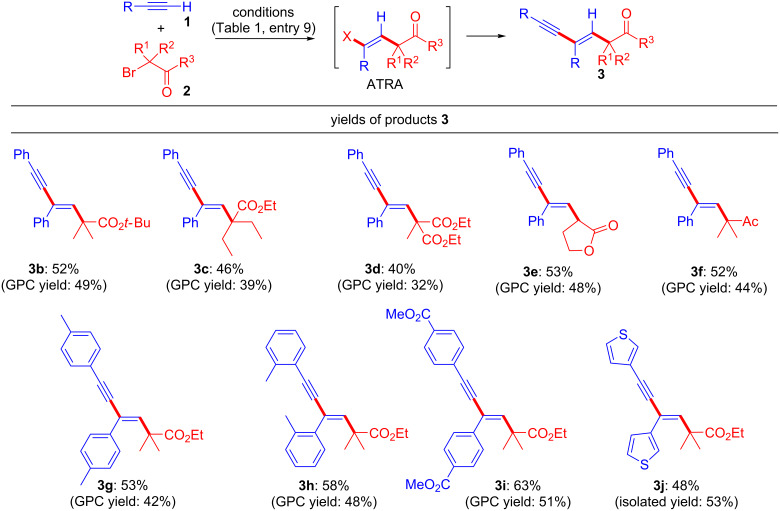
Substrate scope of **1** and **2**. ^a^Conducted at 80 °C for 24 h in MeCN with CuBr (10 mol %), 1,10-Phen (20 mol %), Cs_2_CO_3_ (4.0 equiv), NaI (2.0 equiv), **1** (3.0 equiv) and **2** (1.0 equiv). Yields were determined by ^1^H NMR analysis.

Under the optimized conditions, the reactivities of alkynes **1** and α-bromocarbonyl compounds **2** were examined (Figure 1). The two-step tandem alkyne transformation produced various 1,3-enynes **3** with concomitant formation of ATRA adducts as side-products. It was very difficult to separate the products by silica-gel column chromatography; therefore, we are reporting the ^1^H NMR and GPC yields of **3**. (The pure products were obtained by gel permeation chromatography (GPC).) For example, α-bromocarbonyl compounds **2** possessing various degrees of steric bulkiness (ethyl groups) at the carbonyl α-position or a *tert*-butyl ester group reacted with **1a** to produce **3b** and **3c** in moderate yields. Bromomalonate, bromolactone, and bromoketone derivatives resulted in the formation of **3d**, **3e**, and **3f** in 40%, 53%, and 52% yield, respectively. *Ortho* and *meta* substituted arylalkynes **1** reacted with **2a** to produce **3g** and **3h**, respectively. An arylalkyne **1** possessing an electron-withdrawing group (ester) yielded **3i** without affecting the reactivity of **2**. Sulfur functional groups tend to decrease the catalytic activity of copper salts, but thienyl-substituted alkyne **1** reacted with **2a** to produce **3j** in 48% yield.

**Scheme 2:**
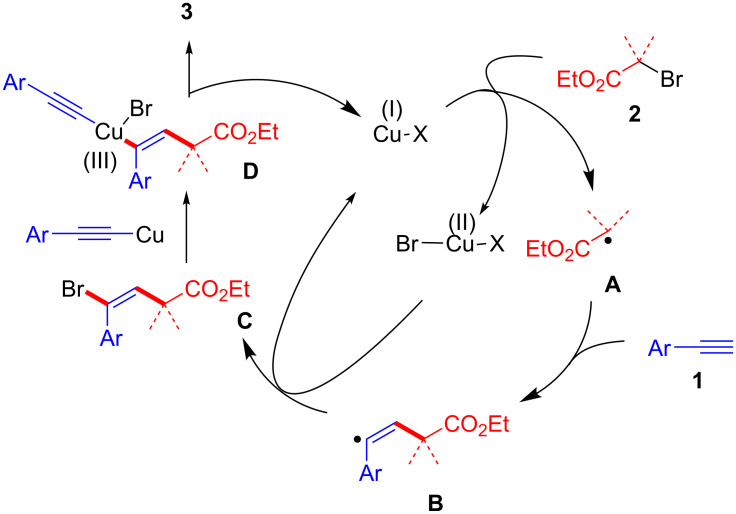
Proposed mechanism.

**Scheme 3:**
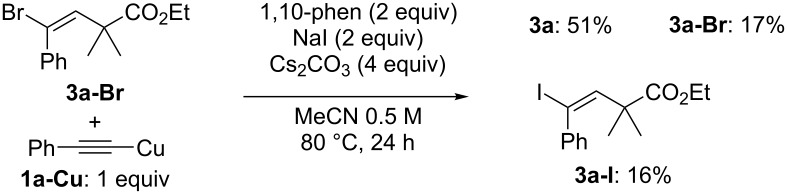
Control experiment.

Although the exact reaction mechanism is currently unclear, one possibility involves a radical pathway including cross-coupling with an alkynyl copper species (Scheme 2). After the generation of **A**, addition of **A** to **1** takes place to give the radical intermediate **B**. This then reacts with the Cu(II) species to produce intermediate **C**, with concomitant formation of a Cu(I) species. The brominated intermediate **C** undergoes a cross-coupling reaction with the alkynyl copper species to give the desired product **3**. We have detected brominated intermediate **C** during the reaction. We have also examined the reaction between **3a-Br** (intermediate **C**) and the alkynyl copper species (**1a**-**Cu**) (Scheme 3). The result showed that **3a-Br** reacted with the alkynyl copper species to produce the desired product **3a** in reasonable yield. This reaction should be a Sonogashira coupling without a Pd catalyst [23–24,30].

**Scheme 4:**
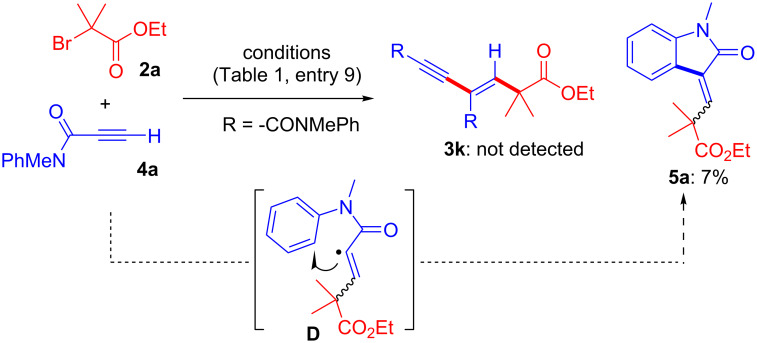
Reaction of **2a** and **4a**.

Interestingly, if the reaction of **2a** and electron-deficient alkyne **4a** was performed under the conditions shown in Table 1, entry 9, the C–H cyclized product **5a** was obtained instead of **3k**. In this case, an alkyl radical addition followed by C–H cyclization via an alkenyl radical intermediate could be occur (Scheme 4). A Pd-catalyzed cascade reaction (C–C bond formation/C–H cyclization process) of *N*-arylpropynamide **4** for the preparation of indolinone derivatives **5** was previously reported by Li’s group [34–35]. In another report on C–H cyclization by Lei’s group, Ni-catalyzed aromatic C–H alkylation occurs via a radical reaction [36]. Both cases were helpful in our development of the current Cu-catalyzed cascade C–H cyclization system.

**Figure 2:**
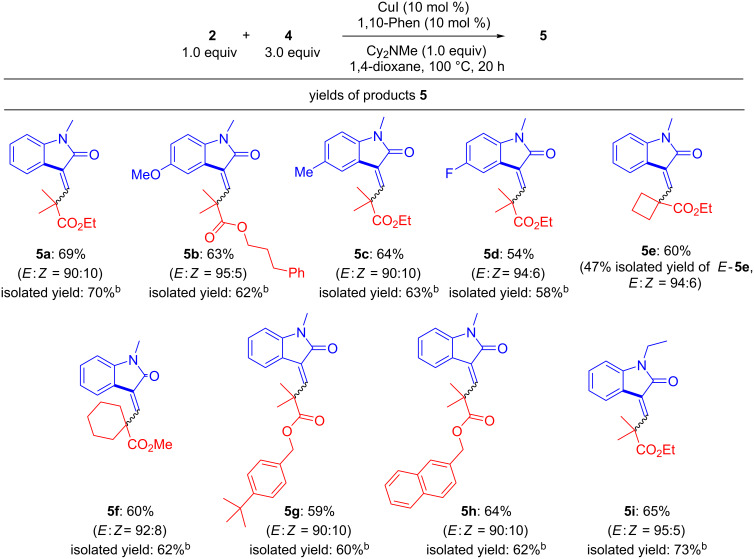
Substrate scope of **2** and **4**. ^a^Conducted at 100 °C for 20 h in 1,4-dioxane with CuI (10 mol %), 1,10-Phen (10 mol %), Cy_2_NMe (1.0 equiv), **2** (1.0 equiv) and **4** (3.0 equiv). Yields were determined by ^1^H NMR analysis. ^b^Including *E* and *Z* isomers.

After careful optimization, we found that CuI, 1,10-Phen, Cy_2_NMe as a base, and 1,4-dioxane were effective for obtaining the best yields of products **5** (Figure 2). In this examination, product isolation was difficult because of the formation of stereoisomers (*E* and *Z* stereoisomers of **5**). The yields shown in Figure 2 were including isomers (We also put yield of pure *E*-**5** as an NMR yield.). Although the chemical yields of **5** were moderate, the stereoselectivities in this reaction were good (the major stereoisomers of **5** produced were *E*). We tested compounds **2** possessing acyclic and cyclic structures and compounds **4** possessing MeO, F, and *N*-Et moieties as substrates for the reaction; however, no big differences were observed. The C–H cyclized products were not obtained from the reaction of compound **4** if it did not possess an alkyl substituent on the nitrogen atom of the amide bond.

## Conclusion

In summary, we have developed two types of tandem reactions catalyzed by a copper salt. The reaction of 2-bromocarbonyl compounds and aryl-substituted alkynes underwent alkyl radical addition at a C–C triple bond followed by Sonogashira coupling to produce 1,3-enyne compounds. On the other hand, the reaction with alkyne possessing a carboxamide moiety underwent tandem alkyl radical addition at the C–C triple bond followed by C–H coupling to produce indolinone derivatives. These results could suggest new aspects of alkyne transformations in a copper catalyzed alkyl radical reaction system.

## Supporting Information

File 1 Experimental procedures, compound characterization data, and NMR spectra.
