# Supplementary material for: Controlling alkyne reactivity by means of a copper-catalyzed radical reaction system for the synthesis of functionalized quaternary carbons
Source: Beilstein J Org Chem. 2020 Mar 26;16:502–8. doi: 10.3762/bjoc.16.45 (PMC7113556; doi:10.3762/bjoc.16.45)

BEILSTEIN JOURNAL OF ORGANIC CHEMISTRY

Supporting Information

for

Controlling alkyne reactivity by means of a copper-catalyzed radical reaction system for the synthesis of functionalized quaternary carbons

Goki Hirata, Yu Yamane, Naoya Tsubaki, Reina Hara and Takashi Nishikata

Beilstein J. Org. Chem. **2020,** *16,* 502–508. doi:10.3762/bjoc.16.45

Experimental procedures, compound characterization data, and NMR spectra

License and Terms: This is a supporting information file under the terms of the Creative Commons Attribution License (http://creativecommons.org/licenses/by/4.0). Please note that the reuse, redistribution and reproduction in particular requires that the authors and source are credited.
The license is subject to the *Beilstein Journal of Organic Chemistry* terms and conditions: (https://www.beilstein-journals.org/bjoc)

Table of Contents

1.	General information	S2
2.	Synthesis and analytical data of 3	S3
3.	Synthesis and analytical data of 5	S8
4.	Reference	S13
5.	Spectral data for new compounds	S14

1. General information.

All reactions were carried out under nitrogen (99.95%) atmosphere. For TLC analyses precoated Kieselgel 60 F254 plates (Merck, 0.25 mm thick) were used; for column chromatography silica gel 60 (Kanto chemical, 63–210 μm) was used. Visualization was accomplished by UV light (254 nm), ^{1}H and ^{13}C NMR spectra were obtained using a JEOL 500 MHz NMR spectrometer. ^{1}H NMR and ^{13}C NMR spectra were obtained in $CDCl_3$ by using $CHCl_3$ (for ^{1}H, δ = 7.26 ppm) and $CDCl_3$ (for ^{13}C, δ = 77.16 ppm) as an internal standard. High resolution mass analyses were obtained using a ACQUITY UPLC/ TOF-MS for EI. Anhydrous toluene and THF were purchased from Kanto Chemical Co., Ltd. Other chemicals obtained from TCI, Sigma-Aldrich and Wako and Copper salts obtained from Sigma-Aldrich and Wako were used directly as supplied.

Table S1. Substrates

Substrates

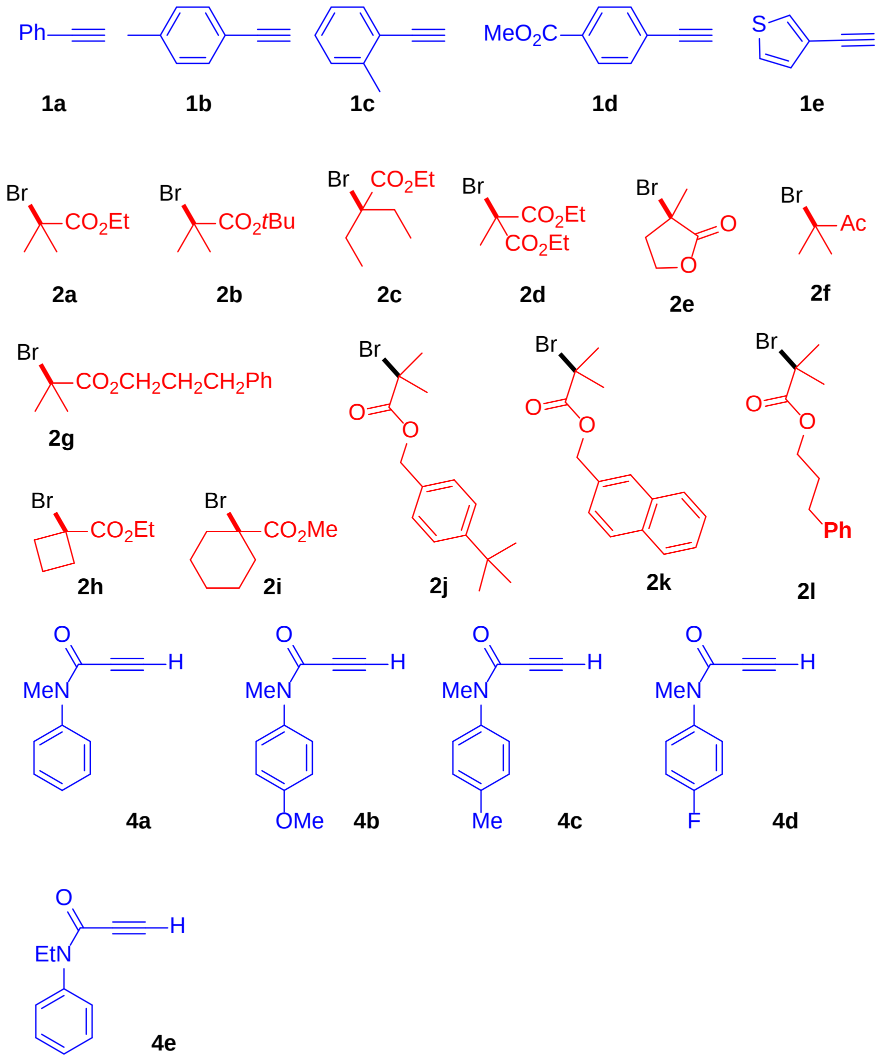

2. Synthesis and analytical data of 3

General Procedure for the reaction: CuBr (0.050 mmol), 1,10-Phen (0.10 mmol), NaI (1.0 mmol) and Cs_2CO_3 (2.0 mmol) were sequentially added to the dram vial equipped with a stir bar and a screw cap. MeCN (1.0 mL), **1** (1.5 mmol) and **2** (0.50 mmol) were added to a dram vial. The resulting mixture vigorously stirred under nitrogen atmosphere (purity 99.95%) for 24 h at 80 °C. After this time, the contents of the flask were filtered through the plug of silica gel with EtOAc as an eluent, and then concentrated by rotary evaporation. The crude residue was purified by flash chromatography and GPC, eluting with EtOAc/hexane to afford the product. The yield was checked by ^{1}H NMR analysis. **3a**, **3d,** **3f** and **3i** are reported compounds[1].

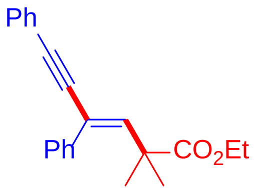

3a

Following the general procedure above, using **2a** (97 mg, 0.5 mmol), **1a** (152 mg, 1.5 mmol), CuBr (10.8 mg, 0.075 mmol), Cs_2CO_3 (656 mg, 2.00 mmol), 1,10-Phen (27.1 mg, 0.15 mmol), NaI (150 mg, 1.0 mmol) and toluene (1.0 mL), the crude residue was purified by flash Chromatography on silica gel with EtOAc/hexane to afford the desired compound **3a** with inseparable side products. The yield was determined by ^{1}H NMR analysis (66%) and purified by GPC (52%); ^{1}H NMR (500 MHz, $CDCl_3$) δ 7.41-7.39 (m, 2H), 7.34-7.27 (m, 8H), 6.26 (s, 1H), 3.60 (q, J = 7.0 Hz, 2H), 1.31 (s, 6H), 1.08 (t, J = 7.0 Hz, 3H); ^{13}C NMR (125 MHz, $CDCl_3$) δ 175.6, 143.3, 137.5, 131.6, 128.8, 128.3, 128.19, 128.12, 127.8, 124.2, 123.3, 91.8, 88.0, 60.7, 44.3, 27.7, 13.9. This compound is a reported compound.[1]

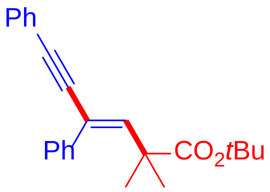

3b

Following the general procedure above, using **2b** (111 mg, 0.5 mmol), **1a** (152 mg, 1.5 mmol), CuBr (10.8 mg, 0.075 mmol), Cs_2CO_3 (656 mg, 2.00 mmol), 1,10-Phen (27.1 mg, 0.15 mmol), NaI (150 mg, 1.0 mmol) and toluene (1.0 mL), the crude residue was purified by flash Chromatography on silica gel with EtOAc/hexane to afford the desired compound **3b** with inseparable side products. The yield was determined by ^{1}H NMR analysis and purified by GPC (52%); ^{1}H NMR (500 MHz, $CDCl_3$) δ 7.42-7.25 (m, 10H), 6.29 (s, 1H), 1.29 (s, 9H), 1.22 (s, 6H); ^{13}C NMR (125 MHz, $CDCl_3$) δ 175.1, 143.2,

138.0, 131.6, 128.8, 128.3, 128.2, 128.1, 127.8, 92.3, 87.6, 80.6, 45.1, 27.8, 27.5; FT-IR (neat, cm^{-1}) 2974, 2930, 2869, 1722, 1488, 1366, 1132; HRMS (ESI-MS) *m/z* [$M+H^+$] Calcd for $C_{24}H_{27}O_2$ 347.2011, found 347.2011.

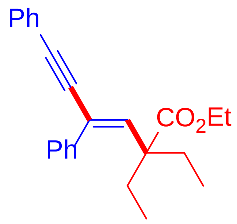

3c

Following the general procedure above, using **2c** (111 mg, 0.5 mmol), **1a** (152 mg, 1.5 mmol), CuBr (10.8 mg, 0.075 mmol), Cs_2CO_3 (656 mg, 2.00 mmol), 1,10-Phen (27.1 mg, 0.15 mmol), NaI (150 mg, 1.0 mmol) and toluene (1.0 mL), the crude residue was purified by flash Chromatography on silica gel with EtOAc/hexane to afford the desired compound **3c** with inseparable side products. The yield was determined by ^1H NMR analysis and purified by GPC (46%); ^1H NMR (500 MHz, $CDCl_3$) δ 7.41-7.39 (m, 2H), 7.34-7.26 (m, 8H), 6.33 (s, 1H), 3.74 (q, J = 7.1 Hz, 2H), 1.74-1.66 (m, 4H), 1.12 (t, J = 7.1 Hz, 3H), 0.82 (t, J = 7.5 Hz, 6H); ^{13}C NMR (125 MHz, $CDCl_3$) δ 174.8, 141.4, 137.9, 131.6, 128.5, 128.3, 128.1, 127.8, 124.8, 123.4, 92.3, 87.6, 60.5, 52.4, 29.2, 14.1, 8.8; FT-IR (neat, cm^{-1}) 2968, 2935, 2876, 1724, 1488, 1441, 1222, 1131; HRMS (EI-MS) *m/z* [$M+H^+$] Calcd for $C_{24}H_{27}O_2$ 347.2011, found 347.2011.

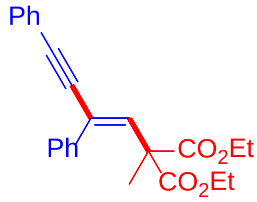

3d

Following the general procedure above, using **2d** (126 mg, 0.5 mmol), **1a** (152 mg, 1.5 mmol), CuBr (10.8 mg, 0.075 mmol), Cs_2CO_3 (656 mg, 2.00 mmol), 1,10-Phen (27.1 mg, 0.15 mmol), NaI (150 mg, 1.0 mmol) and toluene (1.0 mL), the crude residue was purified by flash Chromatography on silica gel with EtOAc/hexane to afford the desired compound **3d** with inseparable side products. The yield was determined by ^1H NMR analysis and purified by GPC (40%); ^1H NMR (500 MHz, $CDCl_3$) δ 7.41-7.39 (m, 2H), 7.35-7.27 (m, 8H), 6.74 (s, 1H), 3.99-3.92 (m, 2H), 3.89-3.83 (m, 2H), 1.55 (s, 3H), 1.16 (t, J = 7.2 Hz, 6H); ^{13}C NMR (125 MHz, $CDCl_3$) δ 170.5, 136.8, 136.1, 131.6, 128.8, 128.3, 128.16, 128.13, 126.1, 123.2, 91.5, 89.0, 61.7, 55.7, 23.3, 13.9. This compound is a reported compound.[1]

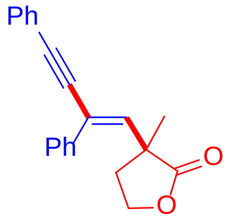

3e

Following the general procedure above, using **2e** (90 mg, 0.5 mmol), **1a** (152 mg, 1.5 mmol), CuBr (10.8 mg, 0.075 mmol), Cs_2CO_3 (656 mg, 2.00 mmol), 1,10-Phen (27.1 mg, 0.15 mmol), NaI (150 mg, 1.0 mmol) and toluene (1.0 mL), the crude residue was purified by flash Chromatography on silica gel with EtOAc/hexane to afford the desired compound **3e** with inseparable side products. The yield was determined by ^1H NMR analysis and purified by GPC (53%); ^1H NMR (500 MHz, $CDCl_3$) δ 7.40-7.34 (m, 7H), 7.29-7.27 (m, 3H), 6.59 (s, 1H), 4.09-4.02 (m, 2H), 2.08-2.02 (m, 1H), 1.66-1.62 (m, 1H), 1.41 (s, 6H); ^{13}C NMR (125 MHz, $CDCl_3$) δ 180.0, 139.4, 137.7, 131.6, 128.7, 128.5, 128.4, 128.3, 128.2, 126.3, 123.1, 91.0, 89.5, 65.2, 44.7, 35.3, 24.7; FT-IR (neat, cm^{-1}) 2970, 2910, 2870, 1764, 1487, 1442, 1088, 1027; HRMS (EI-MS) *m/z* $[M+H^+]$ Calcd for $C_{21}H_{19}O_2$ 303.1385, found 303.1388.

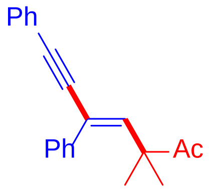

3f

Following the general procedure above, using **2f** (82 mg, 0.5 mmol), **1a** (152 mg, 1.5 mmol), CuBr (10.8 mg, 0.075 mmol), Cs_2CO_3 (656 mg, 2.00 mmol), 1,10-Phen (27.1 mg, 0.15 mmol), NaI (150 mg, 1.0 mmol) and toluene (1.0 mL), the crude residue was purified by flash Chromatography on silica gel with EtOAc/hexane to afford the desired compound **3f** with inseparable side products. The yield was determined by ^1H NMR analysis and purified by GPC (52%); ^1H NMR (500 MHz, $CDCl_3$) δ 7.42-7.40 (m, 2H), 7.35-7.25 (m, 6H), 6.34 (s, 1H), 1.93 (s, 2H), 1.22 (s, 6H); ^{13}C NMR (125 MHz, $CDCl_3$) δ 210.2, 143.3, 137.1, 131.6, 128.8, 128.39, 128.35, 128.2, 91.7, 88.42, 50.8, 26.5, 26.3. This compound is a reported compound.[1]

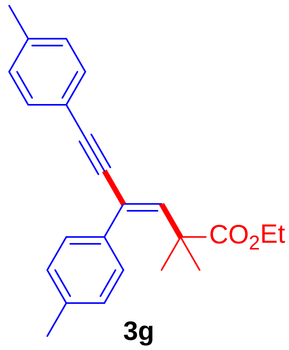

3g

Following the general procedure above, using **2a** (97 mg, 0.5 mmol), **1b** (174 mg, 1.5 mmol), CuBr (10.8 mg, 0.075 mmol), Cs_2CO_3 (656 mg, 2.00 mmol), 1,10-Phen (27.1 mg, 0.15 mmol), NaI (150 mg, 1.0 mmol) and toluene (1.0 mL), the crude residue was purified by flash Chromatography on silica gel with EtOAc/hexane to afford the desired compound **3g** with inseparable side products. The yield was determined by ^{1}H NMR analysis and purified by GPC (53%); ^{1}H NMR (500 MHz, $CDCl_3$) δ 7.30 (d, *J* = 8.1 Hz, 2H), 7.20 (d, *J* = 8.1 Hz, 2H), 7.14 (d, *J* = 7.9 Hz, 2H), 7.08 (d, *J* = 7.9 Hz, 2H), 6.23 (s, 1H), 3.65 (q, *J* = 7.1 Hz, 2H), 2.34 (s, 3H), 2.33 (s, 3H), 1.31 (s, 6H), 1.10 (t, *J* = 7.1 Hz, 3H); ^{13}C NMR (125 MHz, $CDCl_3$) δ 175.8, 142.7, 138.2, 137.5, 134.7, 131.5, 129.0, 128.7, 128.6, 124.4, 120.3, 91.4, 88.0, 60.6, 44.2, 27.7, 21.5, 21.3, 13.9; FT-IR (neat, cm^{-1}) 2975, 2921, 2868, 1727, 1507, 1133; HRMS (EI-MS) *m/z* [M+H$^+$] Calcd for $C_{24}H_{27}O_2$ 347.2011, found 347.2011.

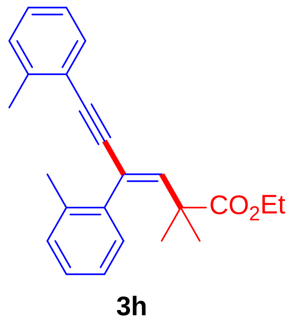

3h

Following the general procedure above, using **2a** (97 mg, 0.5 mmol), **1c** (174 mg, 1.5 mmol), CuBr (10.8 mg, 0.075 mmol), Cs_2CO_3 (656 mg, 2.00 mmol), 1,10-Phen (27.1 mg, 0.15 mmol), NaI (150 mg, 1.0 mmol) and toluene (1.0 mL), the crude residue was purified by flash Chromatography on silica gel with EtOAc/hexane to afford the desired compound **3h** with inseparable side products. The yield was determined by ^{1}H NMR analysis and purified by GPC (58%); ^{1}H NMR (500 MHz, $CDCl_3$) δ 7.26-7.15 (m, 4H), 7.09-7.04 (m, 4H), 6.22 (s, 1H), 3.61 (q, *J* = 7.1 Hz, 2H), 2.34 (s, 3H), 2.30 (s, 3H), 1.30 (m, 2H), 1.09 (t, *J* = 7.2 Hz, 3H); ^{13}C NMR (125 MHz, $CDCl_3$) δ 175.7, 143.1, 137.9, 137.6, 137.4, 132.2, 129.4, 129.0, 128.6, 128.5, 128.2, 128.0, 125.8, 124.4, 123.2, 91.6, 88.1, 60.6, 44.3, 27.7, 21.5,

21.2, 13.9; FT-IR (neat, cm^{-1}) 2975, 2922, 2868, 1727, 1443, 1133; HRMS (EI-MS) *m/z* [$M+H^+$] Calcd for $C_{24}H_{27}O_2$ 347.2011, found 347.2011.

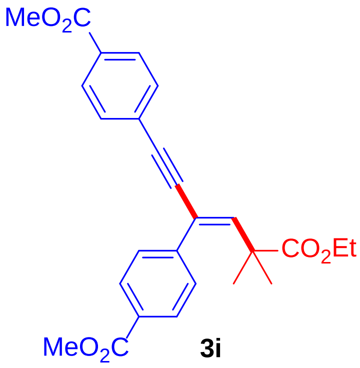

3i

Following the general procedure above, using **2a** (97 mg, 0.5 mmol), **1d** (240 mg, 1.5 mmol), CuBr (10.8 mg, 0.075 mmol), Cs_2CO_3 (656 mg, 2.00 mmol), 1,10-Phen (27.1 mg, 0.15 mmol), NaI (150 mg, 1.0 mmol) and toluene (1.0 mL), the crude residue was purified by flash Chromatography on silica gel with EtOAc/hexane to afford the desired compound **3i** with inseparable side products. The yield was determined by ^1H NMR analysis and purified by GPC (63%); ^1H NMR (500 MHz, $CDCl_3$) δ 8.02 (d, *J* = 7.9 Hz, 2H), 7.96 (d, *J* = 7.9 Hz, 2H), 7.45 (d, *J* = 8.0 Hz, 2H), 7.39 (d, *J* = 8.1 Hz, 2H), 6.36 (s, 1H), 3.93 (s, 3H), 3.91 (s, 3H), 3.66 (q, *J* = 7.1 Hz, 2H), 1.31 (s, 6H), (t, *J* = 7.1 Hz, 3H); ^{13}C NMR (125 MHz, $CDCl_3$) δ 175.2, 166.7, 166.5, 145.1, 141.8, 131.4, 129.6, 129.53, 129.52, 128.8, 127.8, 123.1, 93.9, 87.9, 60.8, 52.3, 52.2, 44.4, 27.5, 13.9; FT-IR (neat, cm^{-1}) 2973, 2951, 1717, 1603, 1434, 1270, 1102; HRMS (EI-MS) *m/z* [$M+H^+$] Calcd for $C_{26}H_{27}O_6$ 435.1808, found 435.1808. This compound is a reported compound.[1]

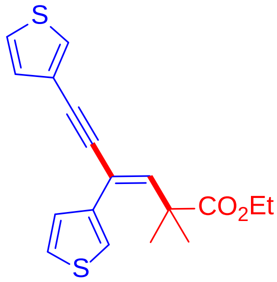

3j

Following the general procedure above, using **2a** (97 mg, 0.5 mmol), **1e** (270 mg, 1.5 mmol), CuBr (10.8 mg, 0.075 mmol), Cs_2CO_3 (656 mg, 2.00 mmol), 1,10-Phen (27.1 mg, 0.15 mmol), NaI (150 mg, 1.0 mmol) and toluene (1.0 mL), the crude residue was purified by flash Chromatography on silica gel

with EtOAc/hexane to afford the desired compound **3j** in 53% yield (87 mg).; ^{1}H NMR (500 MHz, $CDCl_3$) δ 7.41 (dd, *J* = 1.2 and 2.9 Hz, 1H), 7.27-7.24 (m, 2H), 7.18 (dd, *J* = 1.3 and 2.9 Hz, 1H), 7.09 (dd, *J* = 1.2 and 5.0 Hz, 1H), 6.20 (s, 1H), 3.73 (q, *J* = 7.1 Hz, 2H), 1.34 (s, 6H), 1.09 (t, *J* = 7.1 Hz, 3H); ^{13}C NMR (125 MHz, $CDCl_3$) δ 175.8, 143.7, 137.1, 129.9, 128.5, 125.3, 125.1, 124.1, 122.3, 119.1, 90.7, 82.7, 60.8, 44.2, 27.6, 14.0; FT-IR (neat, cm^{-1}) 3104, 2975, 2932, 2868, 1723, 1466, 1132; HRMS (EI-MS) *m/z* [M+H$^+$] Calcd for $C_{18}H_{19}O_2S_2$ 331.0826, found 331.0822.

3. Synthesis and analytical data of 5

General Procedure for the reaction: CuI (10 mol %), 1,10-Phen (10 mol %), Cy_2NMe (1.0 equiv), **2** (1.0 equiv) and **4** (3.0 equiv) were sequentially added to the dram vial equipped with a stir bar and a screw cap. 1,4-dioxane (1.0 mL) was added to a dram vial. The resulting mixture vigorously stirred under nitrogen atmosphere (purity 99.95%) for 20 h at 100 °C. After this time, the contents of the flask were filtered through the plug of silica gel with EtOAc as an eluent, and then concentrated by rotary evaporation. The crude residue was purified by flash chromatography and GPC, eluting with EtOAc/hexane to afford the product. The yield was checked by ^{1}H NMR analysis.

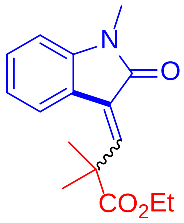

5a

Following the general procedure above, using **2a** (97 mg, 0.5 mmol), **4a** (238.5 mg, 1.5 mmol), CuI (9.5 mg, 0.05 mmol), 1,10-Phen (9 mg, 0.05 mmol), Cy_2NMe (97.6 mg, 0.5 mmol) and 1,4-dioxane (1.0 mL), the crude residue was purified by flash Chromatography on silica gel with EtOAc/hexane to afford the desired compound **5a** with isomers (95 mg, 70%). The desired product yield was determined by ^{1}H NMR analysis (69%) and purified by GPC (*E*-isomer).; ^{1}H NMR (500 MHz, $CDCl_3$) δ 7.30 (d, *J* = 7.6 Hz, 1H), 7.25 (t, *J* = 7.7 Hz, 1H), 7.04 (s, 1H), 6.99 (t, *J* = 7.7 Hz, 1H), 6.80 (d, *J* = 7.7 Hz, 1H), 4.07 (q, *J* = 7.1 Hz, 2H), 3.23 (s, 3H), 1.56 (s, 6H), 1.06 (t, *J* = 7.1 Hz, 3H); ^{13}C NMR (125 MHz, $CDCl_3$) δ 176.0, 168.4, 144.6, 144.2, 129.5, 128.1, 125.2, 122.2, 120.6, 108.1, 61.4, 43.4, 26.3, 26.2, 14.0; FT-IR (neat, cm^{-1}) 2976, 1707, 1607, 1467, 1229, 1137; HRMS (EI-MS) *m/z* [M+H$^+$] Calcd for $C_{16}H_{20}O_3N$ 274.1443, found 274.1443.

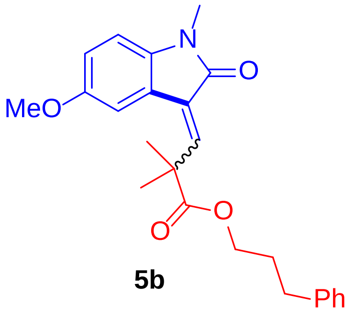

5b

Following the general procedure above, using **2l** (142 mg, 0.5 mmol), **4b** (283.5 mg, 1.5 mmol), CuI (9.5 mg, 0.05 mmol), 1,10-Phen (9 mg, 0.05 mmol), Cy_2NMe (97.6 mg, 0.5 mmol) and 1,4-dioxane (1.0 mL), the crude residue was purified by flash Chromatography on silica gel with EtOAc/hexane to afford the desired compound **5b** with isomers (121 mg, 62%). The desired product yield was determined by ^1H NMR analysis (63%) and purified by GPC (*E*-isomer).; ^1H NMR (500 MHz, $CDCl_3$) δ 7.19 (t, *J* = 7.5 Hz, 2H), 7.13 (t, *J* = 7.4 Hz, 1H), 7.08 (s, 1H), 6.99 (d, *J* = 2.4 Hz, 1H), 6.94 (d, *J* = 6.9 Hz, 2H), 6.82 (dd, *J* = 2.4 and 8.5 Hz, 1H), 6.69 (d, *J* = 8.5 Hz, 1H), 4.04 (q, *J* = 6.3 Hz, 2H), 3.71 (s, 3H), 3.20 (s, 3H), 2.41 (t, *J* = 8.0 Hz, 2H), 1.80-1.74 (m, 2H), 1.59 (s, 6H); ^{13}C NMR (125 MHz, $CDCl_3$) δ 176.1, 168.1, 155.7, 144.7, 141.0, 138.0, 128.6, 128.4, 128.3, 126.0, 121.3, 114.9, 111.7, 108.5, 64.7, 55.9, 43.5, 31.9, 30.0, 26.36, 26.34; FT-IR (neat, cm^{-1}) 2935, 1703, 1592, 1469, 1222, 1129; HRMS (ESI-MS) *m/z* $[M+H^+]$ Calcd for $C_{24}H_{28}O_4N$ 394.2018, found 394.2017.

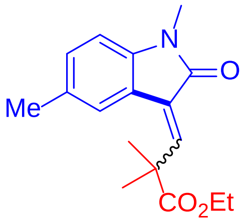

5c

Following the general procedure above, using **2a** (97 mg, 0.5 mmol), **4c** (259.5 mg, 1.5 mmol), CuI (9.5 mg, 0.05 mmol), 1,10-Phen (9 mg, 0.05 mmol), Cy_2NMe (97.6 mg, 0.5 mmol) and 1,4-dioxane (1.0 mL), the crude residue was purified by flash Chromatography on silica gel with EtOAc/hexane to afford the desired compound **5c** with isomers (91 mg, 63%). The desired product yield was determined by ^1H NMR analysis (64%) and purified by GPC (*E*-isomer).; ^1H NMR (500 MHz, $CDCl_3$) δ 7.05 (s, 1H), 7.00 (d, *J* = 7.9 Hz, 1H), 6.94 (s, 1H), 6.62 (d, *J* = 7.9 Hz, 1H), 4.01 (q, *J* = 7.1 Hz, 2H), 3.15 (s, 3H), 2.25 (s, 3H), 1.50 (s, 6H), 1.02 (t, *J* = 7.1 Hz, 3H); ^{13}C NMR (125 MHz, $CDCl_3$) δ 176.0, 168.4, 144.3, 142.0, 131.5, 129.7, 128.3, 126.0, 120.7, 107.8, 61.4, 43.5, 26.4, 26.3, 21.3, 14.1; FT-IR (neat, cm^{-1}) 2977, 1706, 1616, 1488, 1365, 1338, 1255, 1133; HRMS (ESI-MS) *m/z* $[M+H^+]$ Calcd for $C_{17}H_{22}O_3N$ 288.1600, found 288.1600.

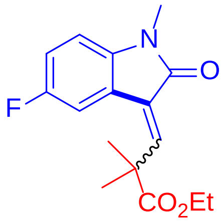

5d

Following the general procedure above, using **2a** (97 mg, 0.5 mmol), **4d** (265.5 mg, 1.5 mmol), CuI (9.5 mg, 0.05 mmol), 1,10-Phen (9 mg, 0.05 mmol), Cy_2NMe (97.6 mg, 0.5 mmol) and 1,4-dioxane (1.0 mL), the crude residue was purified by flash Chromatography on silica gel with EtOAc/hexane to afford the desired compound **5d** with isomers (84 mg, 58%). The desired product yield was determined by ^1H NMR analysis (54%) and purified by GPC (*E*-isomer).; ^1H NMR (500 MHz, $CDCl_3$) δ 7.09 (dd, *J* = 2.5 and 8.9 Hz, 1H), 7.08 (s, 1H), 6.96 (dt, *J* = 2.5 and 8.8 Hz, 1H), 6.70 (dd, *J* = 4.3 and 8.6 Hz, 1H), 4.10 (q, *J* = 7.1 Hz, 2H), 3.21 (s, 3H), 1.55 (s, 6H), 1.10 (t, *J* = 7.1 Hz, 3H); ^{13}C NMR (125 MHz, $CDCl_3$) δ 175.5, 168.1, 158 (d, *J* = 239 Hz), 140.3, 127 (d, *J* = 2.8 Hz), 121 (d, *J* = 8.9 Hz), 115 (d, *J* = 23.7 Hz), 113 (d, *J* = 27 Hz), 108 (d, *J* = 8.3 Hz), 61.5, 43.5, 26.3, 26.2, 14.0; FT-IR (neat, cm^{-1}) 2978, 1708, 1618, 1467, 1138; HRMS (ESI-MS) *m/z* $[M+H^+]$ Calcd for $C_{16}H_{19}O_3NF$ 292.1349, found 292.1349.

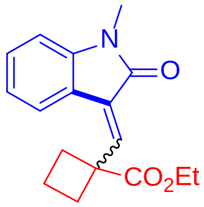

5e

Following the general procedure above, using **2h** (103 mg, 0.5 mmol), **4a** (238.5 mg, 1.5 mmol), CuI (9.5 mg, 0.05 mmol), 1,10-Phen (9 mg, 0.05 mmol), Cy_2NMe (97.6 mg, 0.5 mmol) and 1,4-dioxane (1.0 mL), the crude residue was purified by flash Chromatography on silica gel with EtOAc/hexane to afford the desired compound **5e** (47% pure *E*-compound). The yield was determined by ^1H NMR analysis (60%) and purified by GPC (*E*-isomer).; ^1H NMR (500 MHz, $CDCl_3$) δ 7.31 (d, *J* = 7.5 Hz, 1H), 7.27 (dt, *J* = 1.1 and 7.8 Hz, 1H), 7.25 (s, 1H), 7.00 (dt, *J* = 0.9 and 7.6 Hz, 1H), 6.80 (d, *J* = 7.8 Hz, 1H), 4.10 (q, *J* = 7.1 Hz, 2H), 3.25 (s, 3H), 2.95-2.90 (m, 2H), 2.45-2.40 (m, 2H), 2.08-1.97 (m, 2H), 1.13 (t, *J* = 7.1 Hz, 3H); ^{13}C NMR (125 MHz, $CDCl_3$) δ 173.7, 168.1, 144.0, 142.7, 129.5, 129.1, 125.0, 122.2, 120.3, 108.0, 61.4, 48.1, 33.1, 26.2, 16.2, 14.1; FT-IR (neat, cm^{-1}) 2939, 1706, 1607, 1467, 1377, 1336, 1215, 1094; HRMS (ESI-MS) *m/z* $[M+H^+]$ Calcd for $C_{17}H_{20}O_3N$ 286.1443, found 286.1443.

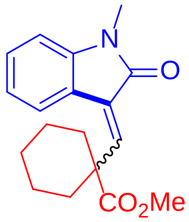

5f

Following the general procedure above, using **2i** (110 mg, 0.5 mmol), **4a** (238.5 mg, 1.5 mmol), CuI (9.5 mg, 0.05 mmol), 1,10-Phen (9 mg, 0.05 mmol), Cy_2NMe (97.6 mg, 0.5 mmol) and 1,4-dioxane (1.0 mL), the crude residue was purified by flash Chromatography on silica gel with EtOAc/hexane to afford the desired compound **5f** with isomers (92 mg, 62%). The desired product yield was determined by ^1H NMR analysis (60%) and purified by GPC (*E*-isomer).; ^1H NMR (500 MHz, $CDCl_3$) δ 7.38 (d, *J* = 7.6 Hz, 1H), 7.27 (t, *J* = 7.7 Hz, 1H), 7.15 (s, 1H), 7.01 (t, *J* = 7.7 Hz, 1H), 6.80 (d, *J* = 7.7 Hz, 1H), 3.57 (s, 3H), 3.24 (s, 3H), 2.10-2.00 (m, 4H), 1.68-1.52 (m, 5H), 0.97-0.88 (m, 1H); ^{13}C NMR (125 MHz, $CDCl_3$) δ 176.0, 168.2, 144.3, 143.0, 129.4, 128.2, 125.3, 122.3, 120.6, 108.1, 52.5, 47.4, 34.0, 26.2, 25.3, 22.3; FT-IR (neat, cm^{-1}) 2931, 1701, 1605, 1464, 1239, 1069; HRMS (ESI-MS) *m*/*z* $[M+H^+]$ Calcd for $C_{18}H_{22}O_3N$ 300.1600, found 300.1602.

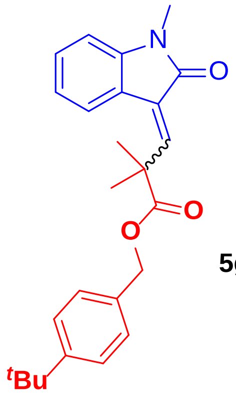

5g

Following the general procedure above, using **2j** (156 mg, 0.5 mmol), **4a** (238.5 mg, 1.5 mmol), CuI (9.5 mg, 0.05 mmol), 1,10-Phen (9 mg, 0.05 mmol), Cy_2NMe (97.6 mg, 0.5 mmol) and 1,4-dioxane (1.0 mL), the crude residue was purified by flash Chromatography on silica gel with EtOAc/hexane to afford the desired compound **5g** with isomers (118 mg, 60%). The desired product yield was determined by ^1H NMR analysis (59%) and purified by GPC (*E*-isomer).; ^1H NMR (500 MHz, $CDCl_3$) δ 7.25-7.22 (m, 4H), 7.06 (d, *J* = 8.2 Hz, 2H), 7.04 (s, 1H), 6.89 (t, *J* = 7.7 Hz, 1H), 6.79 (d, *J* = 7.7

Hz, 1H),5.02 (s, 2H), 3.23 (s, 3H), 1.59 (s, 6H), 1.28 (s, 9H); ^{13}C NMR (125 MHz, $CDCl_3$) δ 175.9, 168.3, 151.2, 144.3, 144.2, 132.5, 129.4, 128.2, 128.0, 125.4, 125.1, 122.3, 120.5, 108.1, 67.1, 43.6, 34.6, 31.3, 26.3, 26.2; FT-IR (neat, cm^{-1}) 2959, 1708, 1608, 1466, 1255, 1134; HRMS (ESI-MS) *m/z* [M+H$^+$] Calcd for $C_{25}H_{30}O_3N$ 392.2226, found 392.2226.

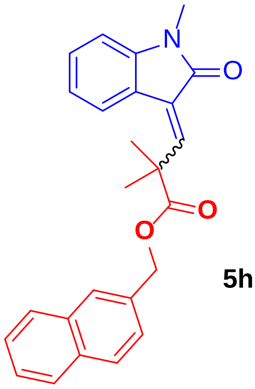

5h

Following the general procedure above, using **4a** (153 mg, 0.5 mmol), **2k** (238.5 mg, 1.5 mmol), CuI (9.5 mg, 0.05 mmol), 1,10-Phen (9 mg, 0.05 mmol), Cy_2NMe (97.6 mg, 0.5 mmol) and 1,4-dioxane (1.0 mL), the crude residue was purified by flash Chromatography on silica gel with EtOAc/hexane to afford the desired compound **5h** with isomers (121 mg, 62%). The desired product yield was determined by ^{1}H NMR analysis (64%) and purified by GPC (*E*-isomer).; ^{1}H NMR (500 MHz, $CDCl_3$) δ 7.76-7.74 (m, 1H), 7.67-7.63 (m, 2H), 7.57 (s, 1H), 7.46-7.42 (m, 2H), 7.19 (d, *J* = 8.1 Hz, 2H), 7.11 (t, *J* = 7.7 Hz, 1H), 7.06 (s, 1H), 6.74 (t, *J* = 7.6 Hz, 1H), 6.70 (d, *J* = 7.6 Hz, 1H), 5.21 (s, 2H), 3.21 (s, 3H), 1.60 (s, 6H); ^{13}C NMR (125 MHz, $CDCl_3$) δ 175.8, 144.1, 144.0, 133.08, 133.04, 132.84, 129.37, 128.3, 128.1, 128.0, 127.6, 127.5, 126.2, 126.1, 125.8, 125.0, 122.1, 120.4, 107.9, 67.3, 43.5, 25.3, 26.2; FT-IR (neat, cm^{-1}) 3053, 2973, 1703, 1607, 1466, 1123; HRMS (ESI-MS) *m/z* [M+H$^+$] Calcd for $C_{26}H_{24}O_3N$ 386.1756, found 386.1756.

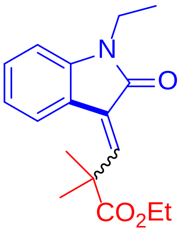

5i

Following the general procedure above, using **4e** (97 mg, 0.5 mmol), **2a** (259.5 mg, 1.5 mmol), CuI (9.5 mg, 0.05 mmol), 1,10-Phen (9 mg, 0.05 mmol), Cy_2NMe (97.6 mg, 0.1 mmol) and 1,4-dioxane (1.0 mL), the crude residue was purified by flash Chromatography on silica gel with EtOAc/hexane to

afford the desired compound **5i** with isomers (105 mg, 73%). The desired product yield was determined by ^{1}H NMR analysis (65%) and purified by GPC (*E*-isomer).; ^{1}H NMR (500 MHz, $CDCl_3$) δ 7.31 (d, *J* = 7.6 Hz, 1H), 7.25 (t, *J* = 7.7 Hz, 1H), 7.04 (s, 1H), 6.98 (t, *J* = 7.7 Hz, 1H), 6.82 (d, *J* = 7.7 Hz, 1H), 4.09 (q, *J* = 7.1 Hz, 2H), 3.79 (q, *J* = 7.2 Hz, 2H), 1.57 (s, 6H), 1.26 (t, *J* = 7.1 Hz, 3H), 1.08 (t, *J* = 7.2 Hz, 3H); ^{13}C NMR (125 MHz, $CDCl_3$) δ 176.0, 167.9, 144.4, 143.3, 129.4, 128.2, 125.3, 122.0, 120.8, 108.2, 61.4, 43.4, 34.7, 26.3, 14.1, 12.8; FT-IR (neat, cm^{-1}) 2976, 1704, 1606, 1466, 1344, 1221, 1138; HRMS (ESI-MS) *m/z* [$M+H^+$] Calcd for $C_{17}H_{22}O_3N$ 288.1600, found 288.1600.

4. Reference

1 C. Che, H. Zheng, G. Zhu, *Org. Lett.* **2015**, *17*, 1617-1620.

5. Spectral charts for new compounds

^{13}C NMR($CDCl_3$)

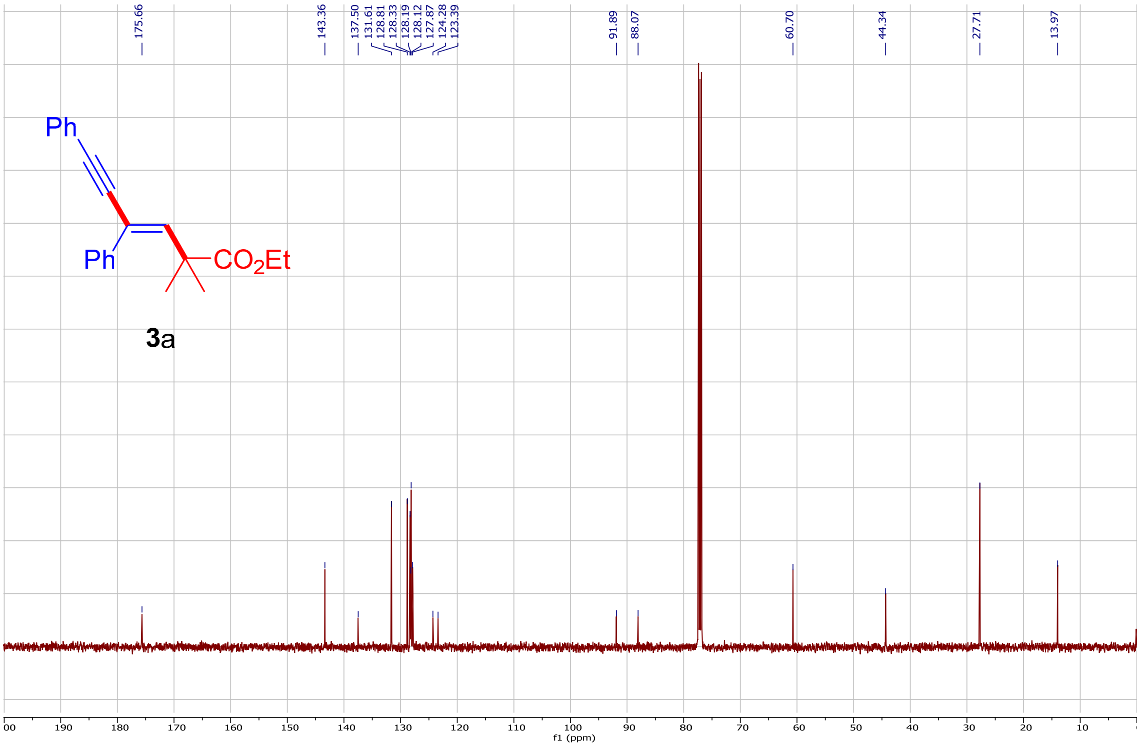

^{1}H NMR($CDCl_3$)

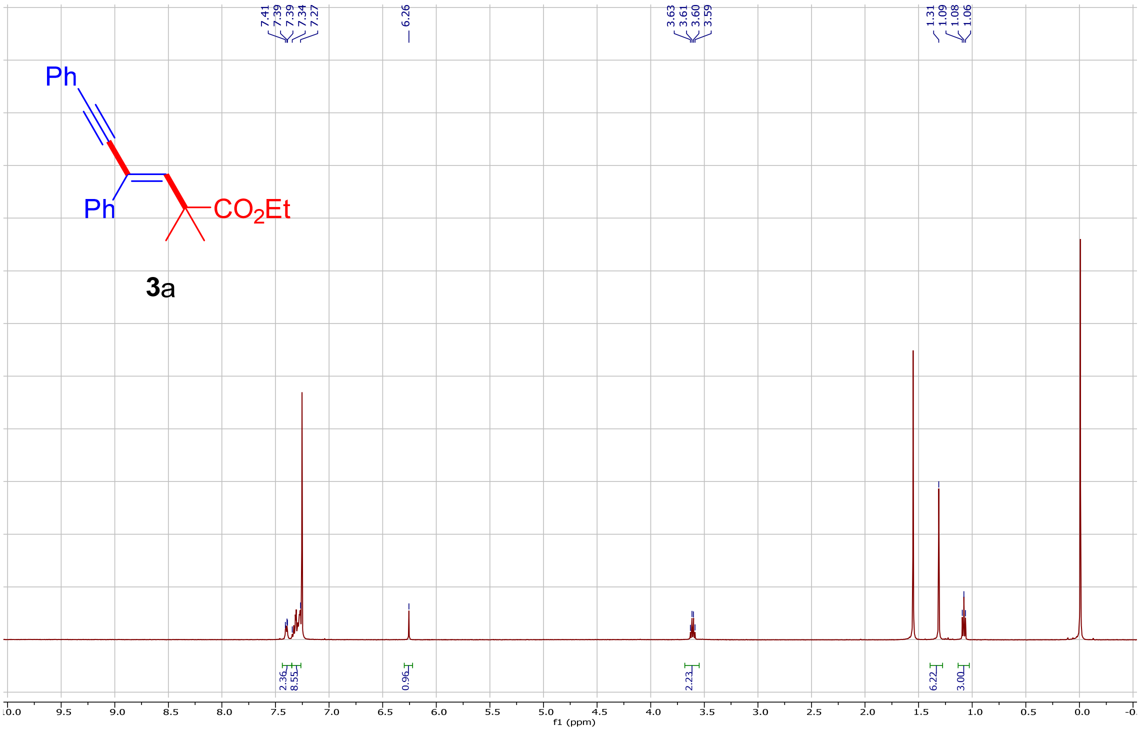

^{13}C NMR($CDCl_3$)

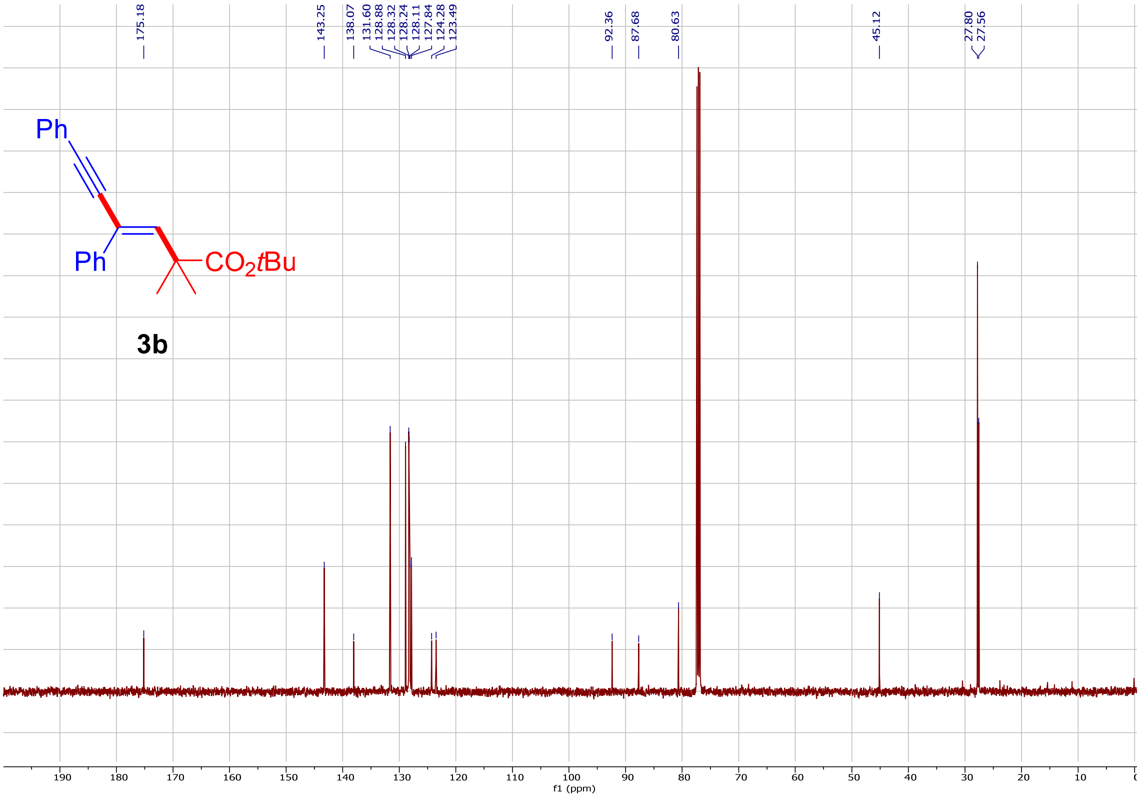

^{1}H NMR($CDCl_3$)

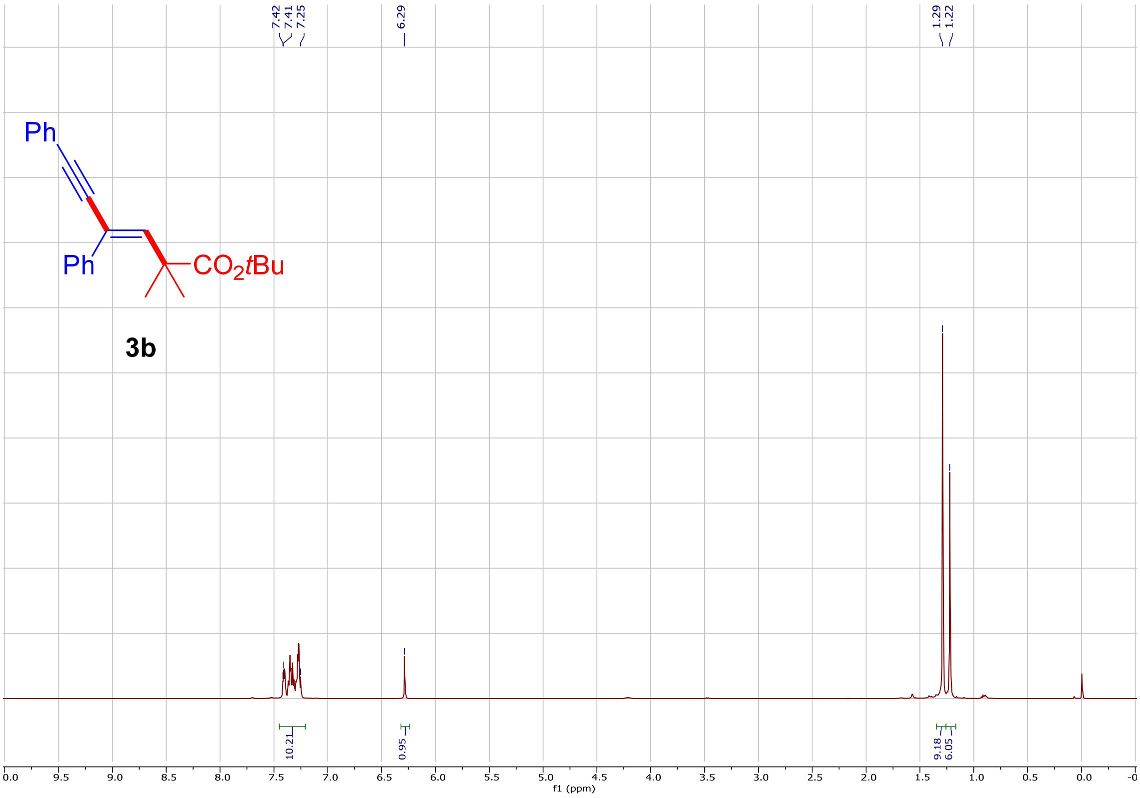

^{13}C NMR(CDCl$_3$)

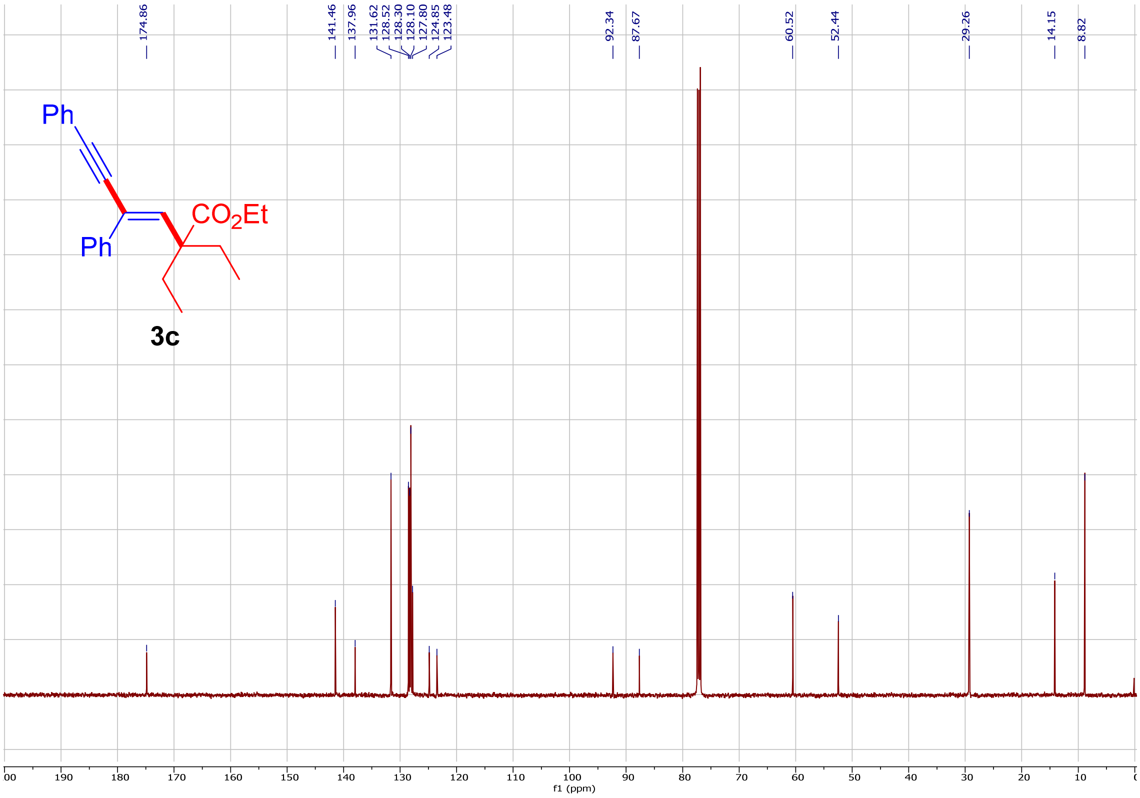

^{1}H NMR(CDCl$_3$)

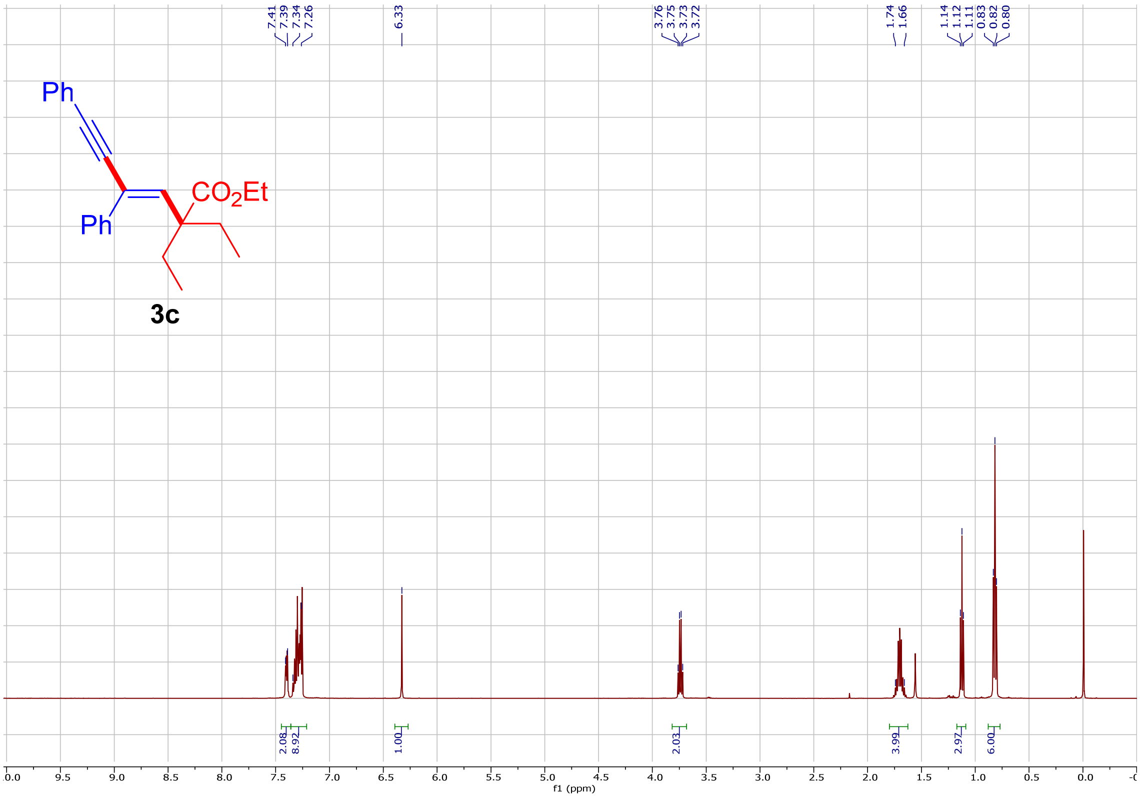

^{13}C NMR($CDCl_3$)

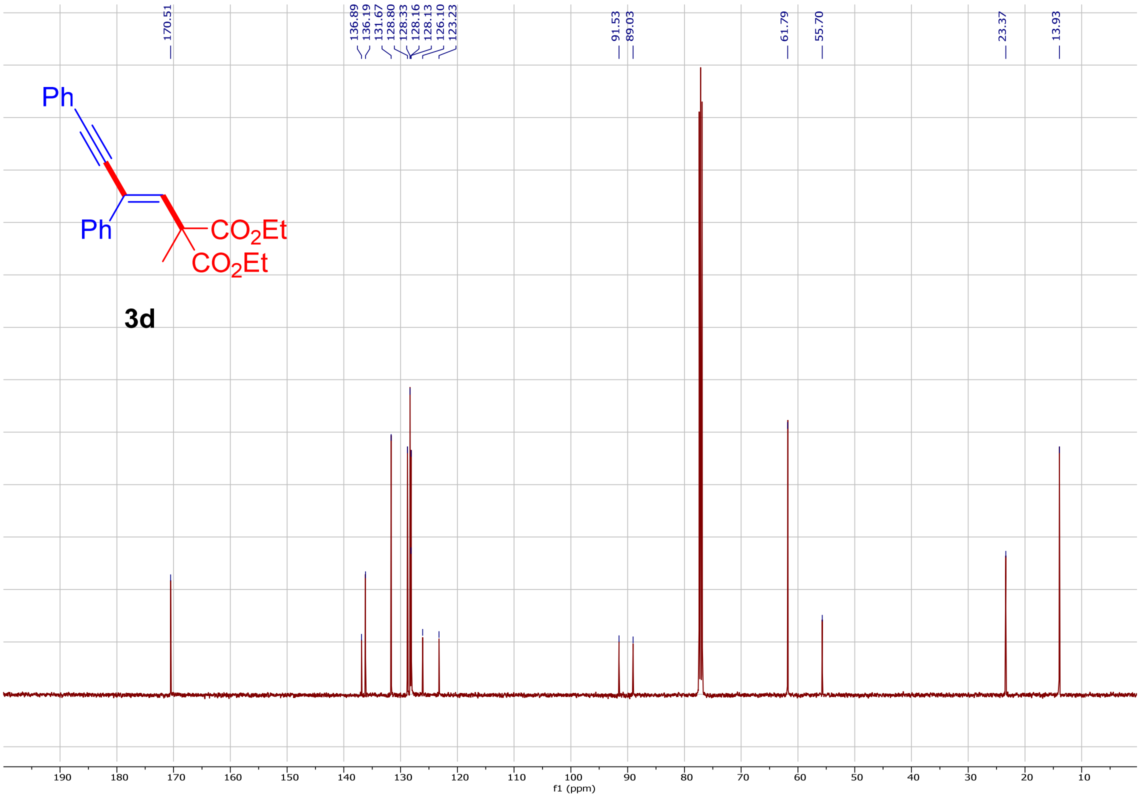

^{1}H NMR($CDCl_3$)

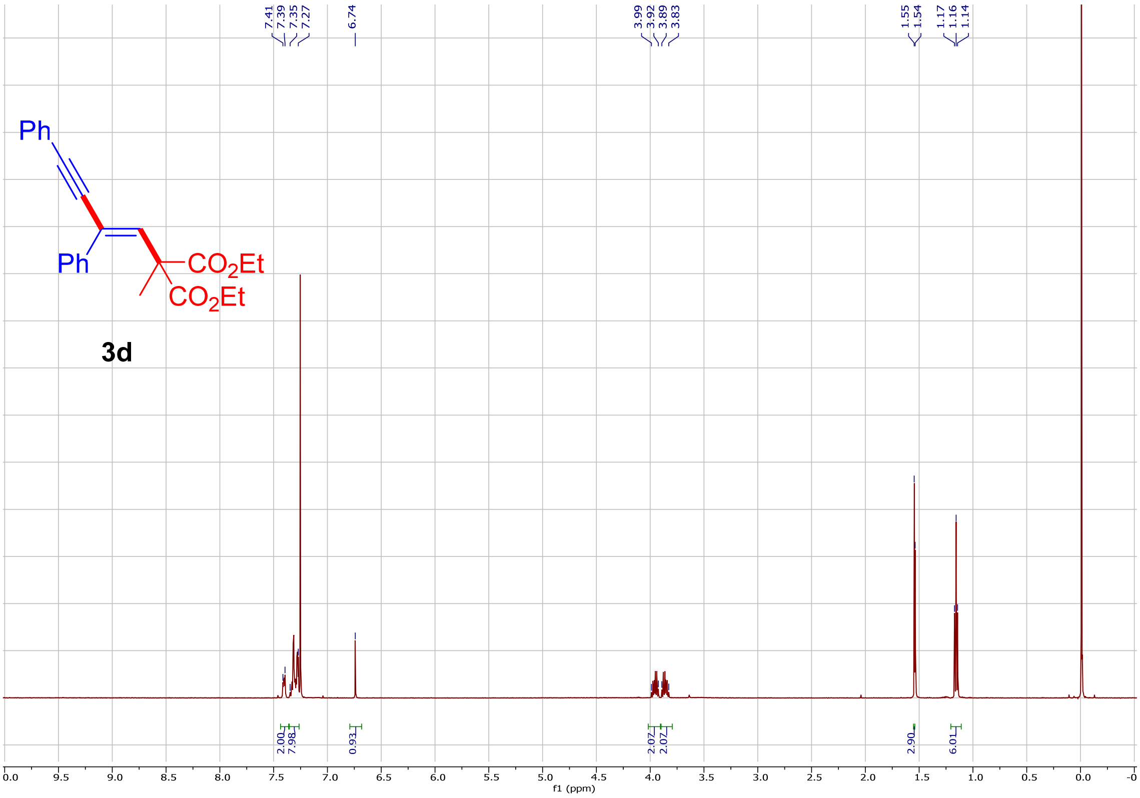

^{13}C NMR($CDCl_3$)

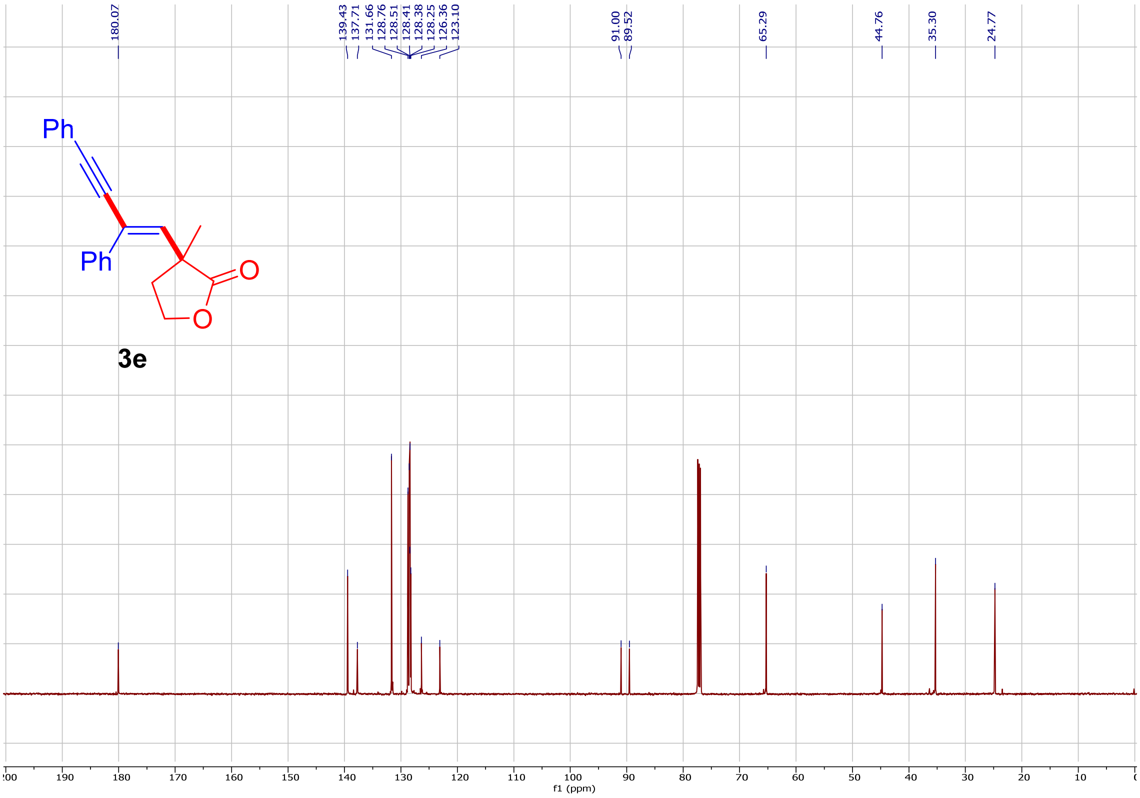

^{1}H NMR($CDCl_3$)

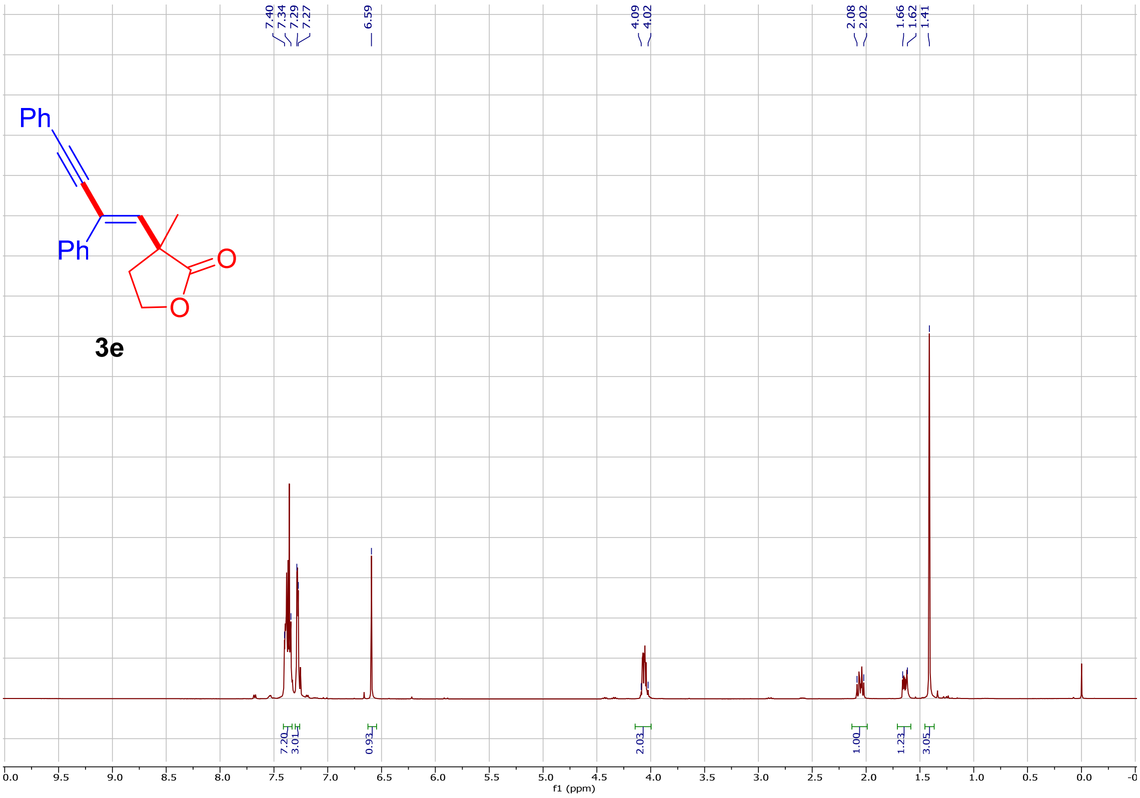

^{13}C NMR(CDCl$_3$)

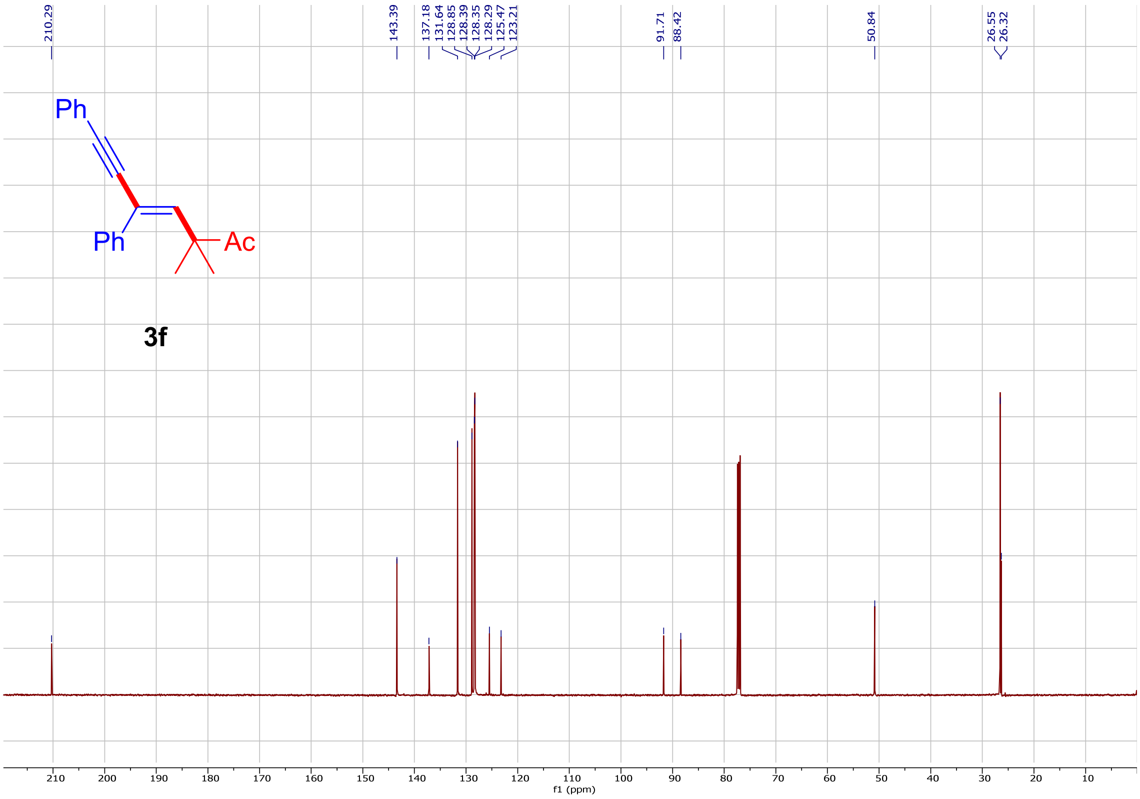

^{1}H NMR(CDCl$_3$)

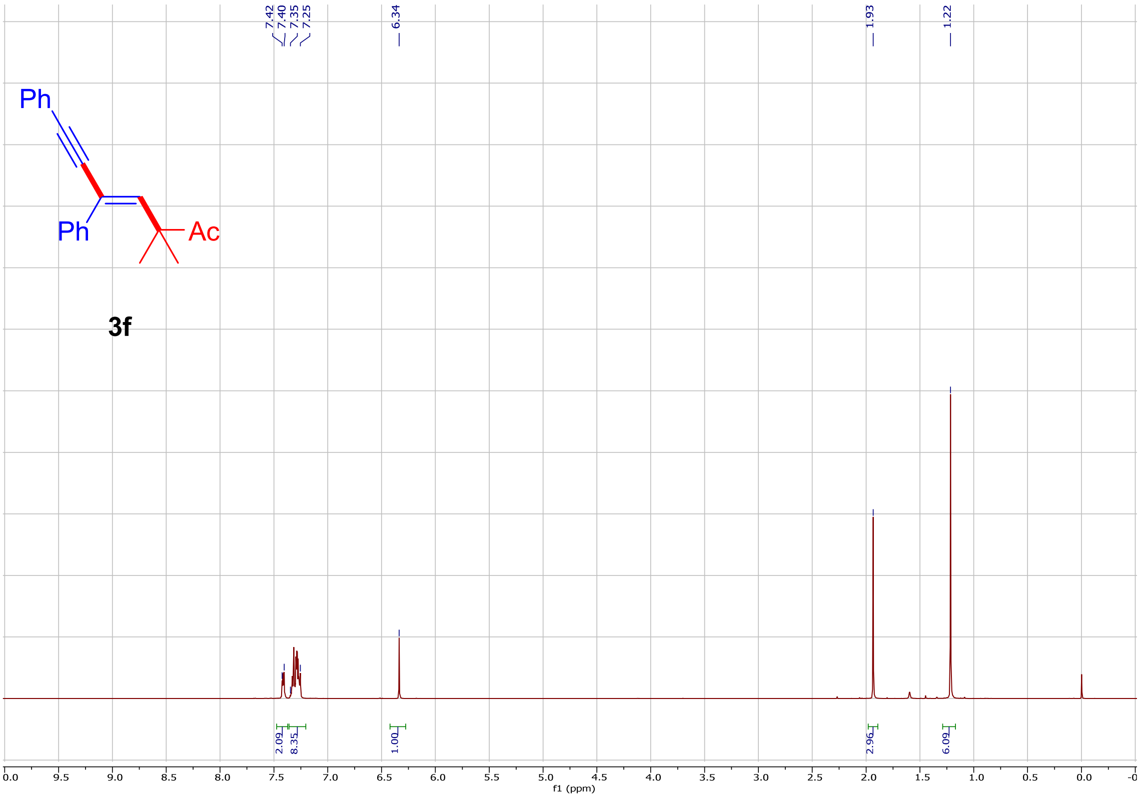

^{13}C NMR($CDCl_3$)

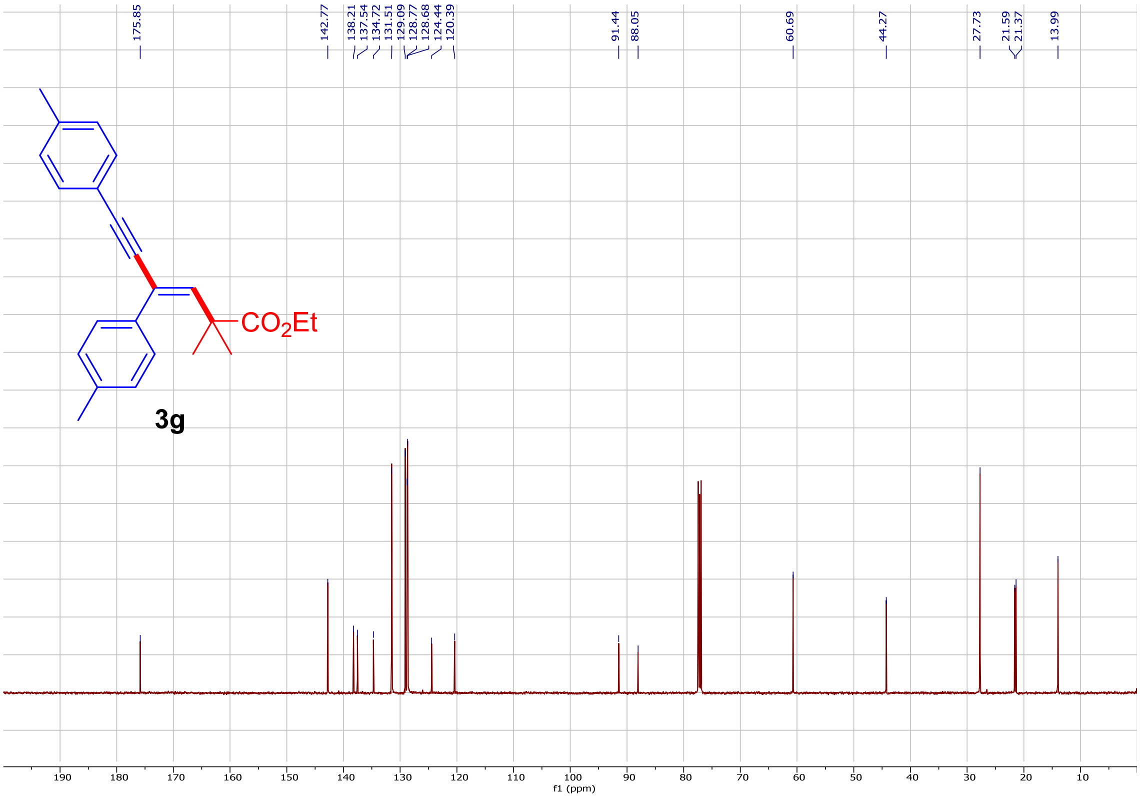

^{1}H NMR($CDCl_3$)

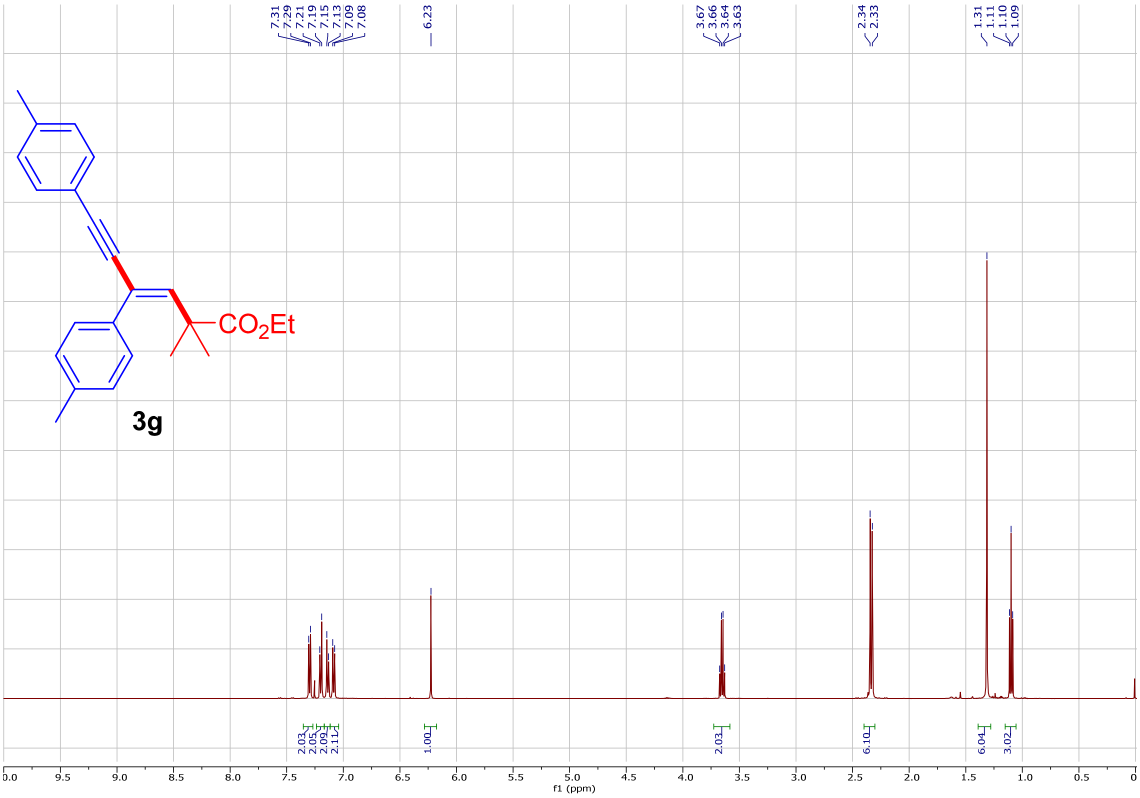

^{13}C NMR($CDCl_3$)

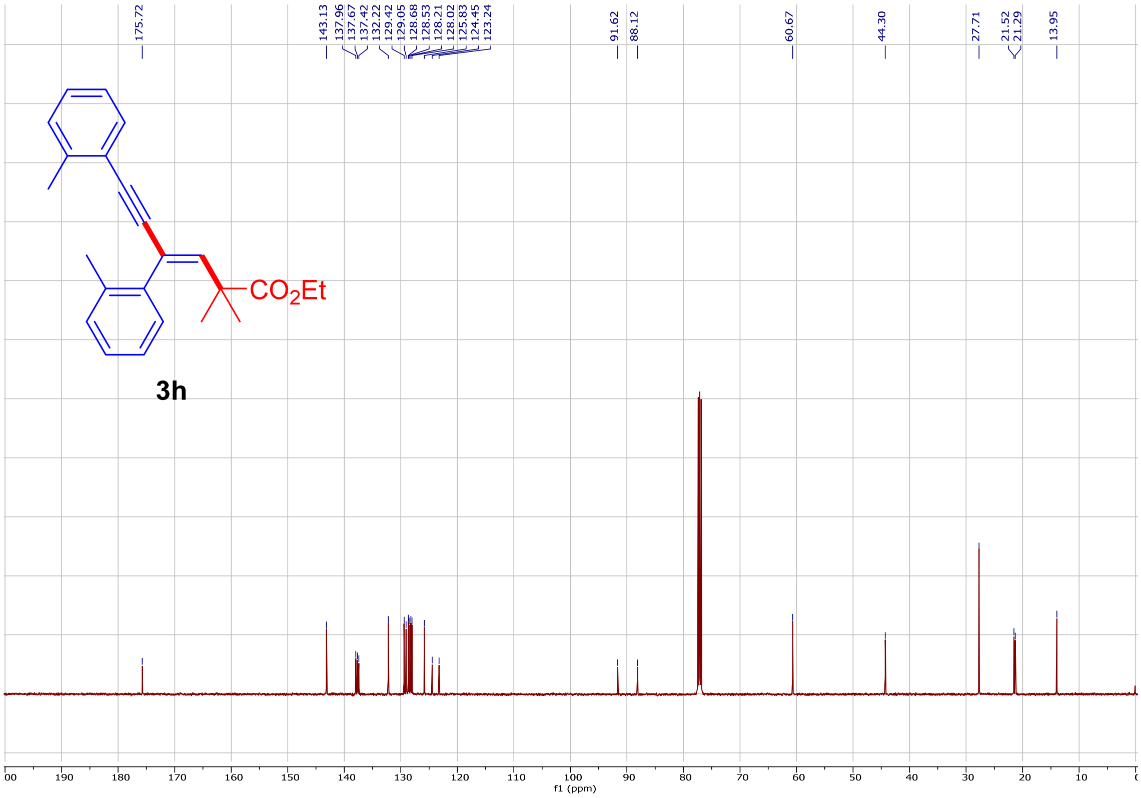

^{1}H NMR($CDCl_3$)

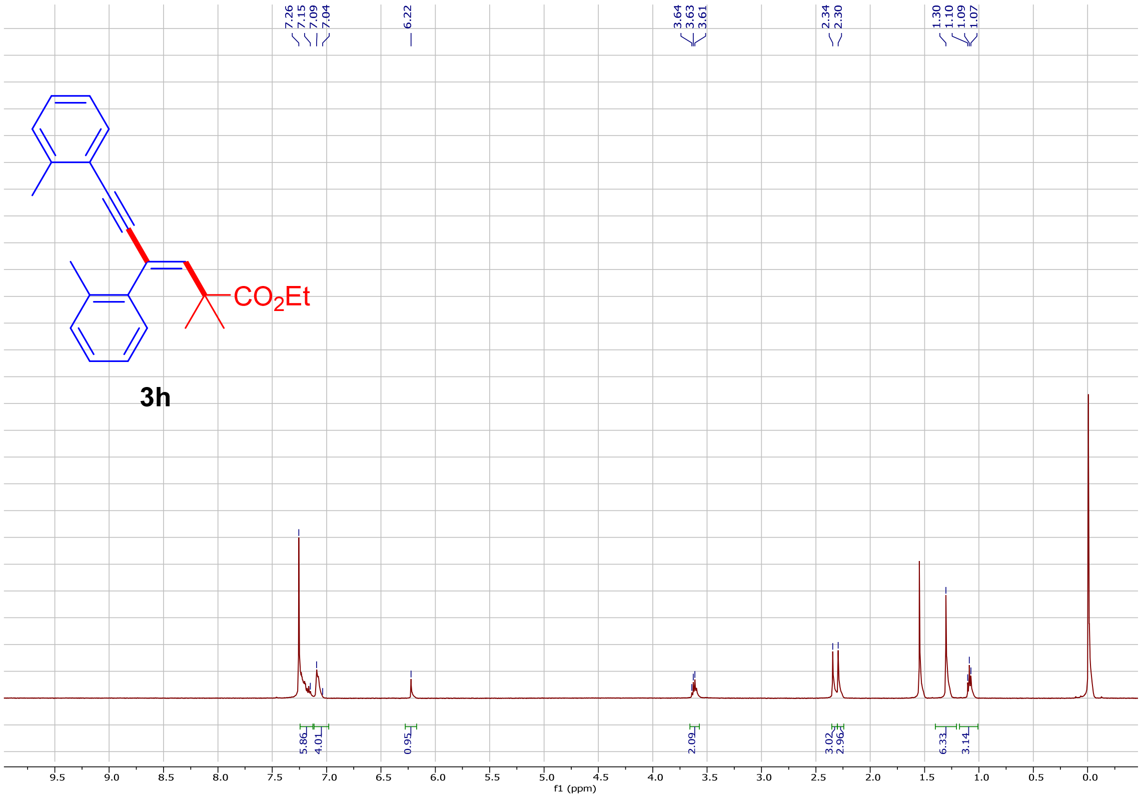

^{13}C NMR(CDCl3)

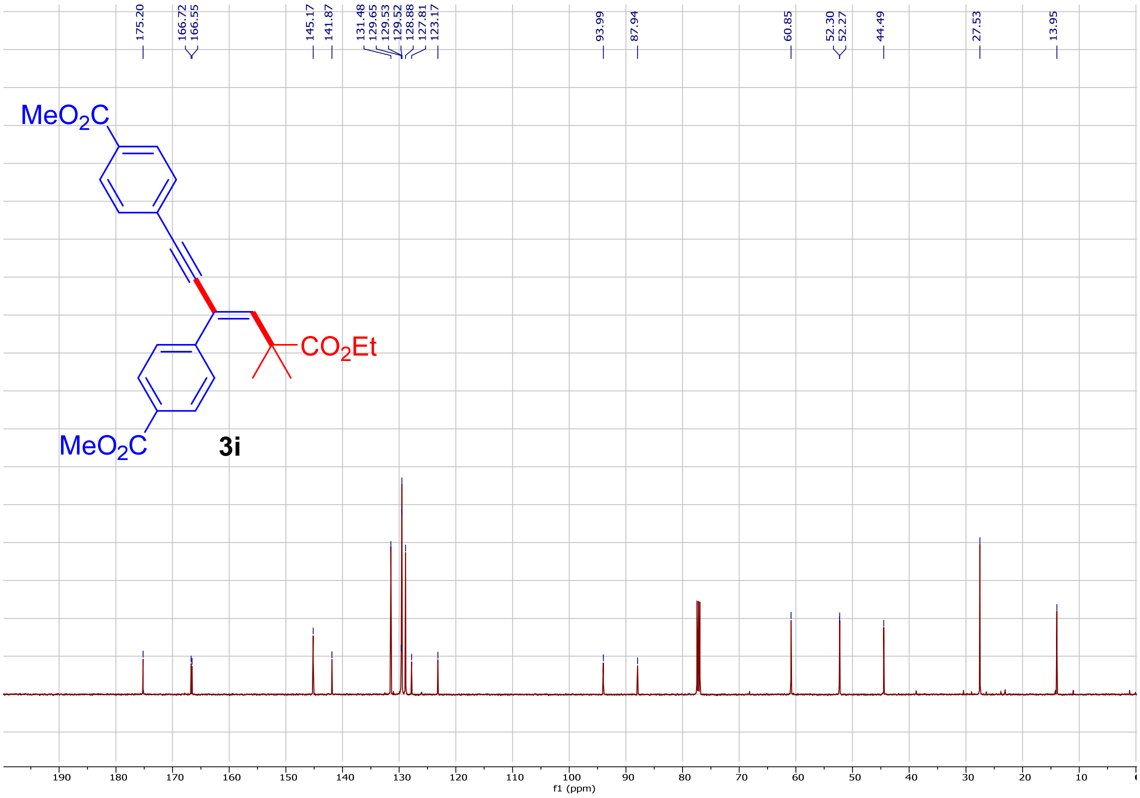

^{1}H NMR(CDCl3)

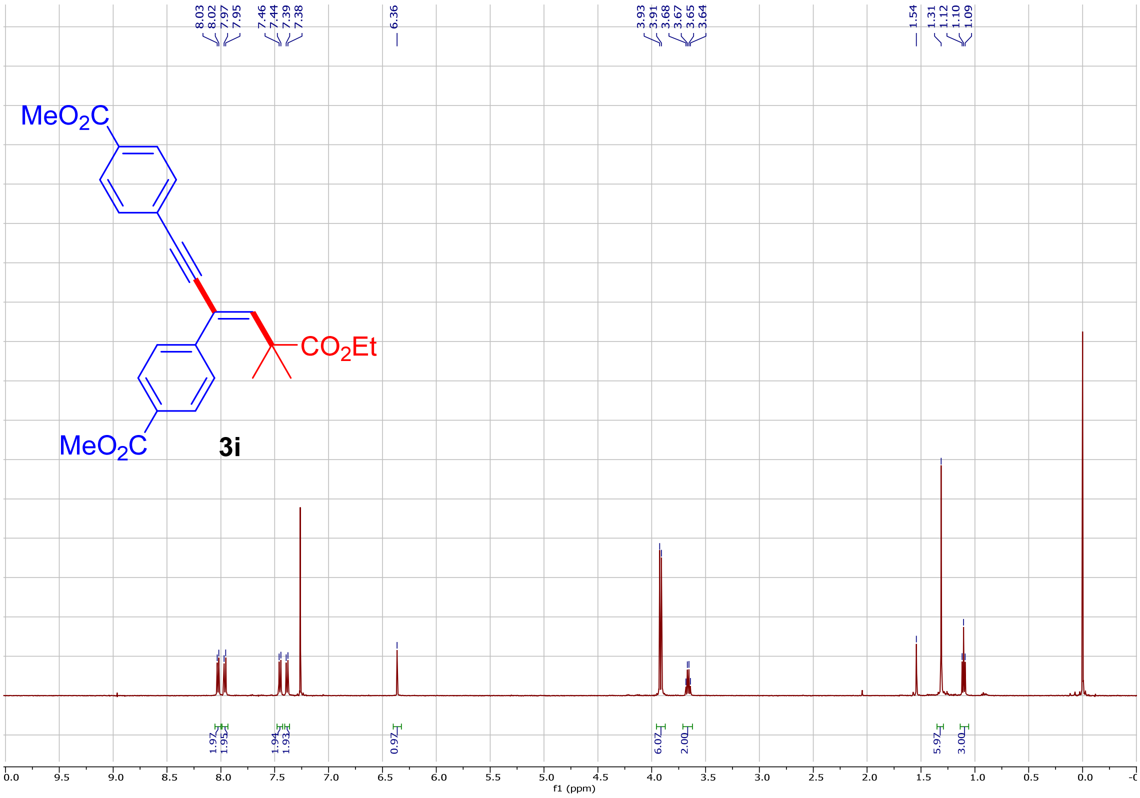

^{13}C NMR($CDCl_3$)

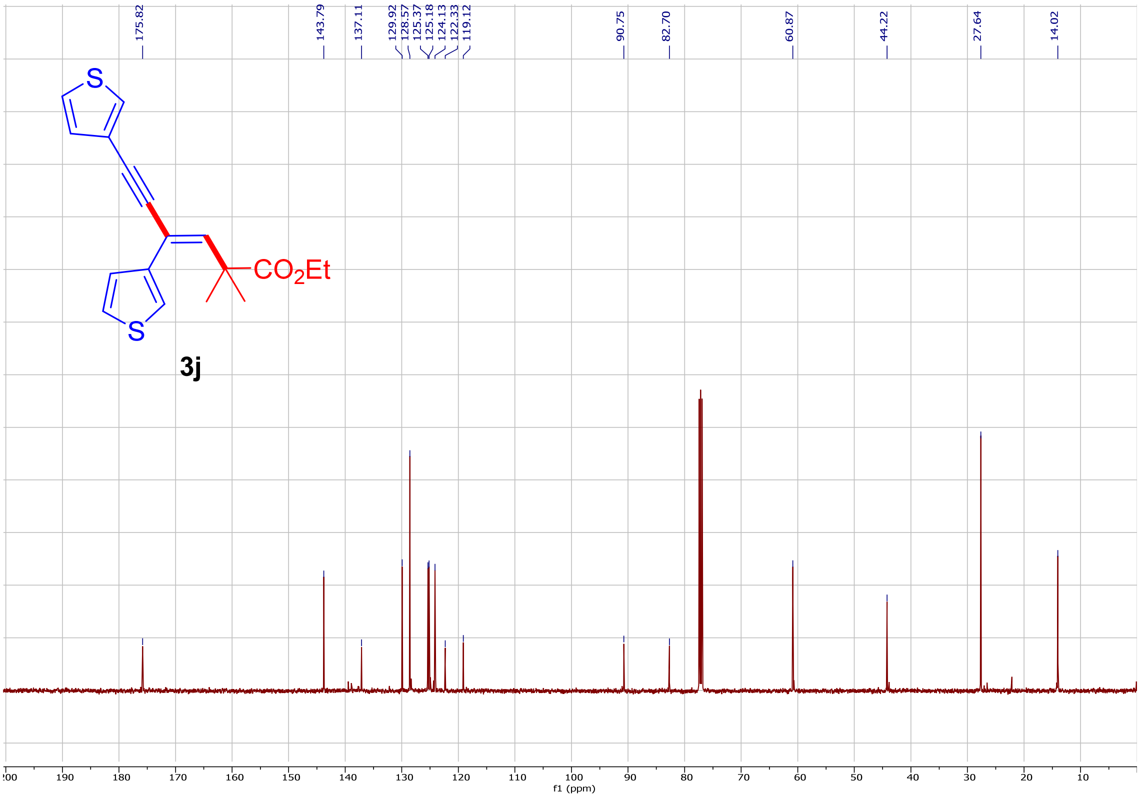

^{1}H NMR($CDCl_3$)

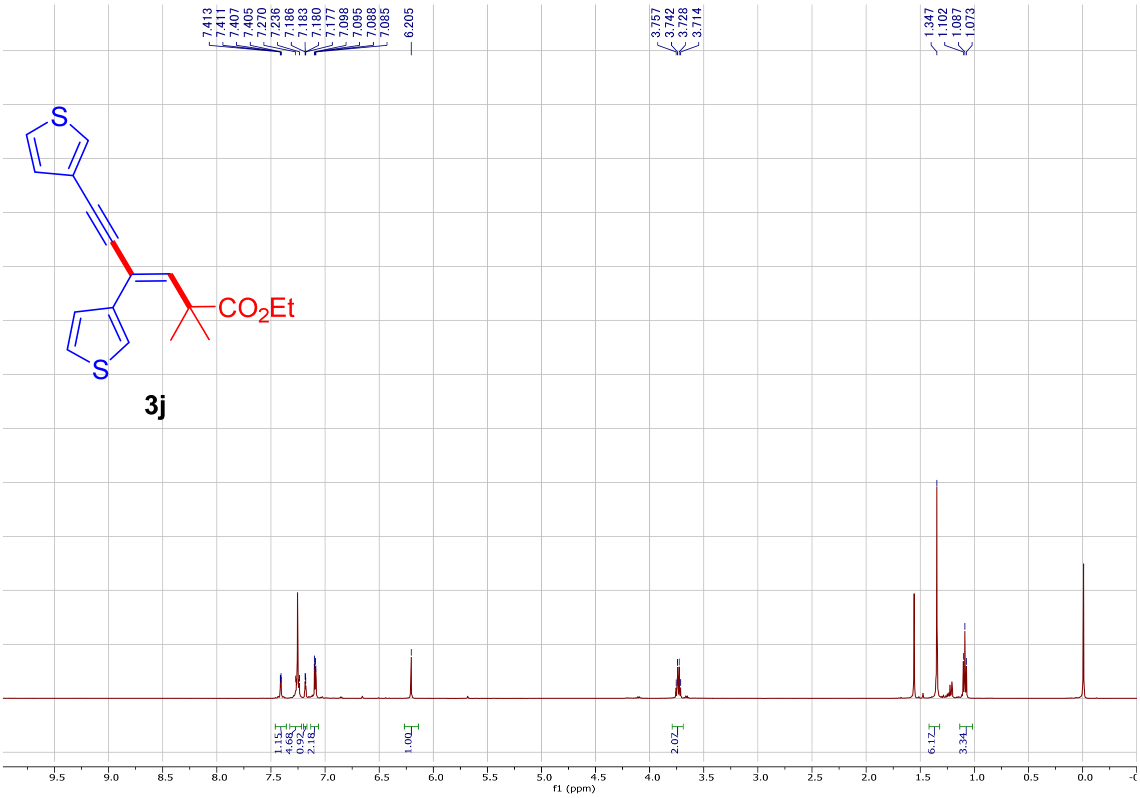

^{13}C NMR($CDCl_3$)

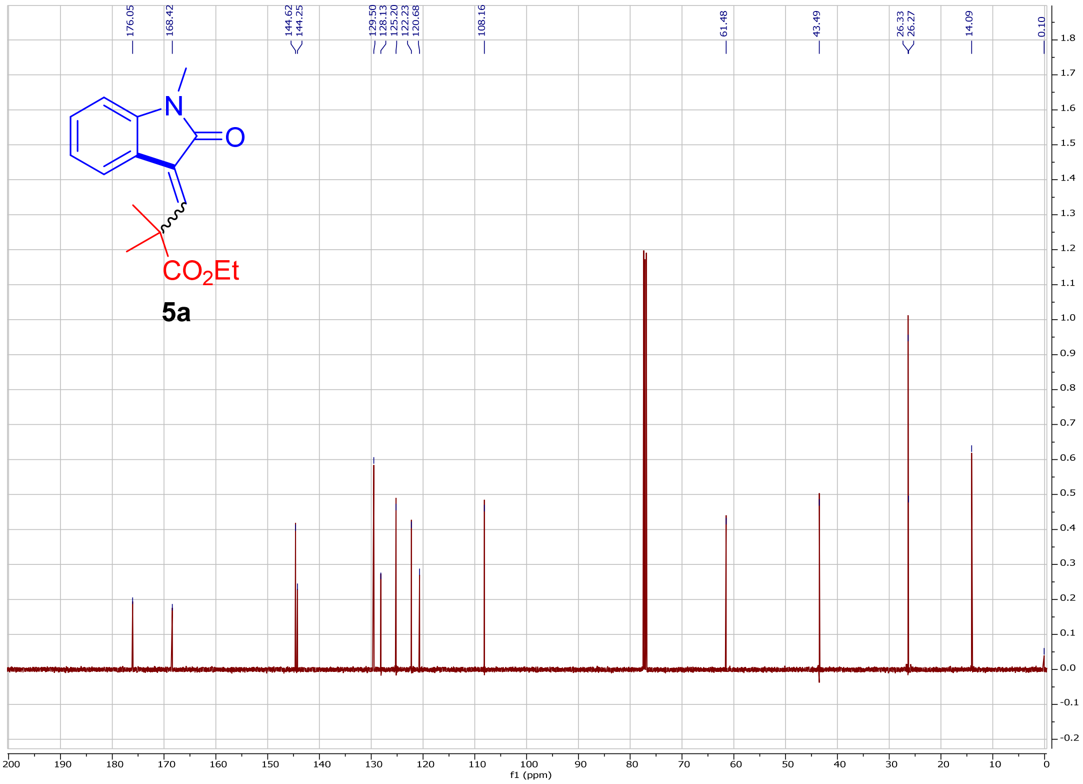

^{1}H NMR($CDCl_3$)

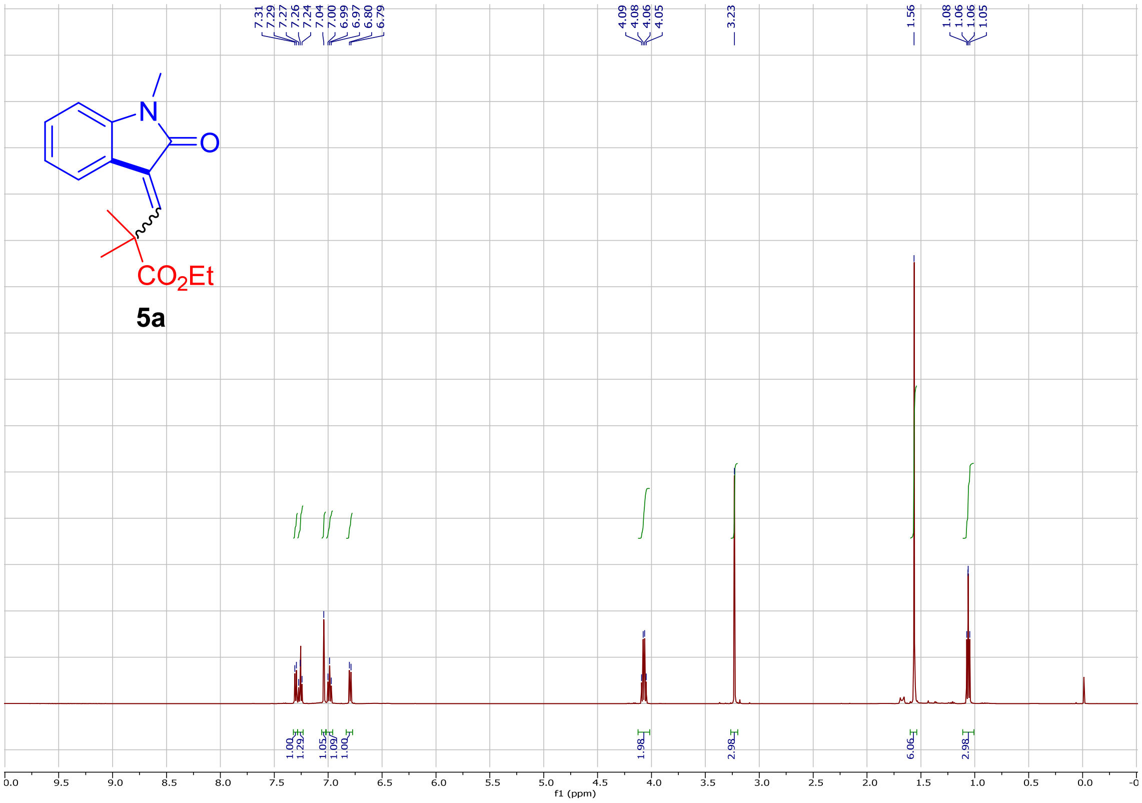

NOESY

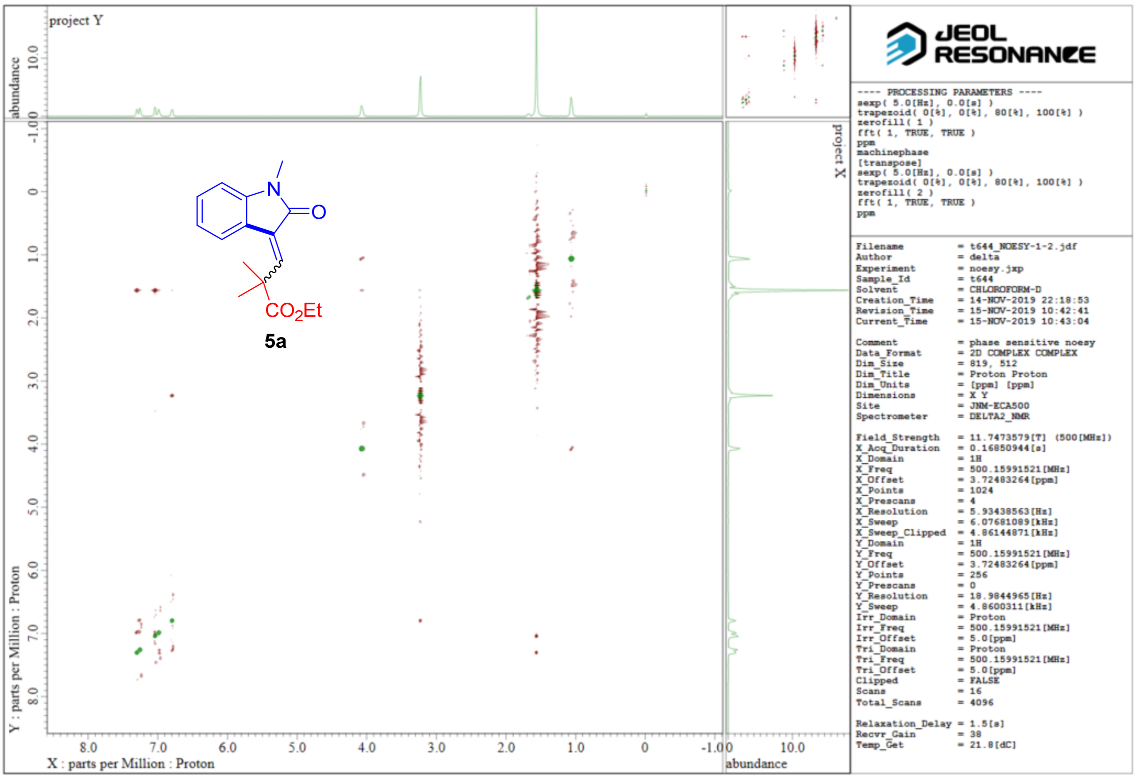
project Y
abundance
project X
JEOL RESONANCE
5a
X : parts per Million : Proton
Y : parts per Million : Proton
Filename = t644_NOESY-1-2.jdf
Author = delta
Experiment = noesy.jxp
Sample_Id = t644
Solvent = CHLOROFORM-D
Creation_Time = 14-NOV-2019 22:18:53
Revision_Time = 15-NOV-2019 10:42:41
Current_Time = 15-NOV-2019 10:43:04
Comment = phase sensitive noesy
Data_Format = 2D COMPLEX COMPLEX
Dim_Size = 819, 512
Dim_Title = Proton Proton
Spectrometer = DELTA2_NMR
Field_Strength = 11.7473579[T] (500[MHz])
Scans = 16
Total_Scans = 4096
Relaxation_Delay = 1.5[s]
Recvr_Gain = 38
Temp_Get = 21.8[dC]

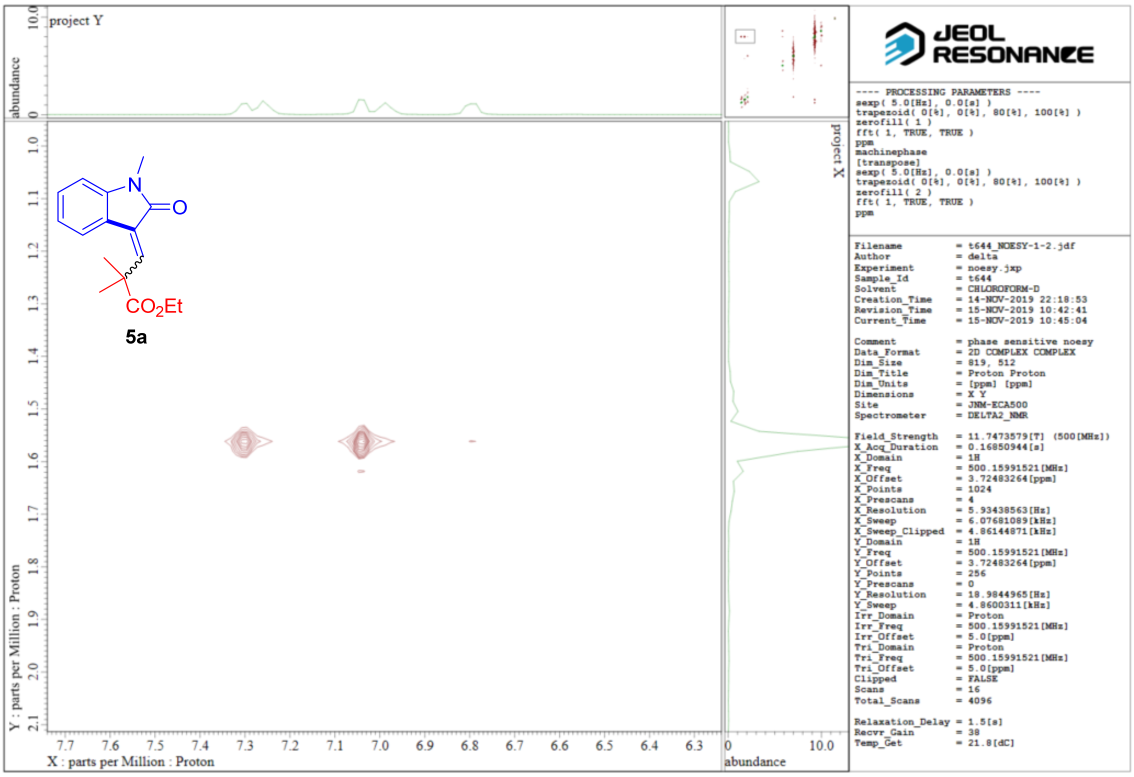
project Y
abundance
project X
JEOL RESONANCE
5a
X : parts per Million : Proton
Y : parts per Million : Proton
Filename = t644_NOESY-1-2.jdf
Current_Time = 15-NOV-2019 10:45:04

^{13}C NMR($CDCl_3$)

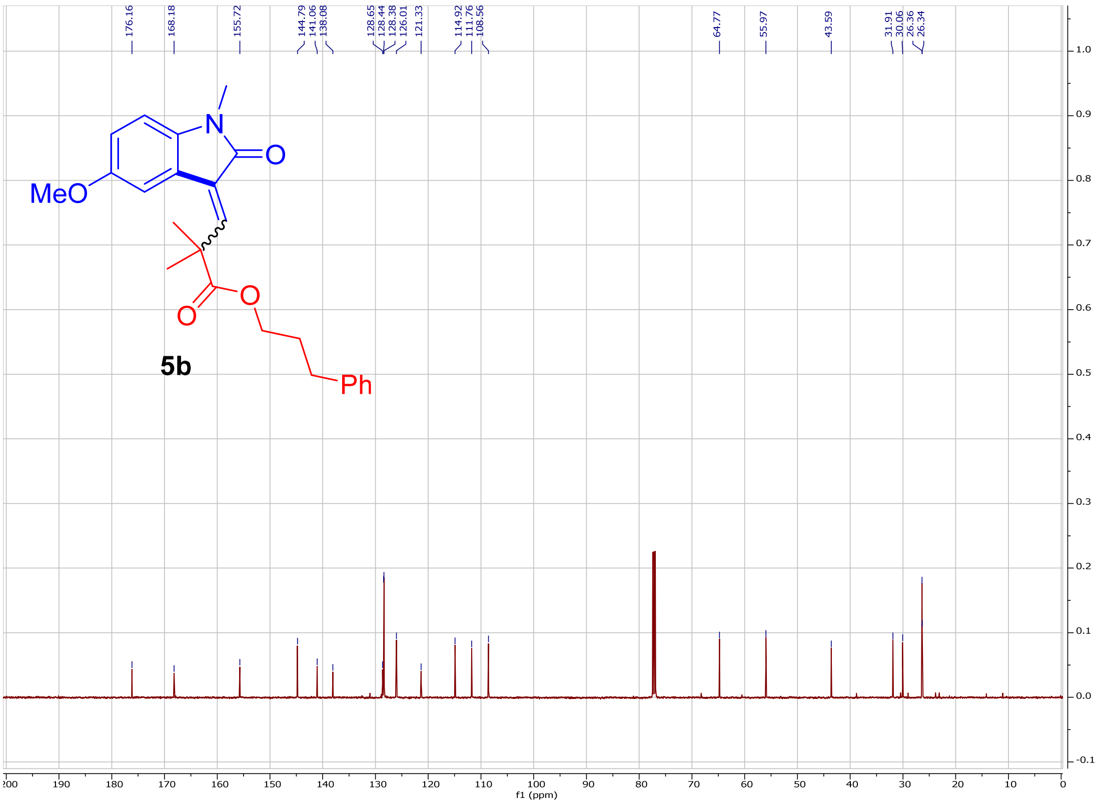

^{1}H NMR($CDCl_3$)

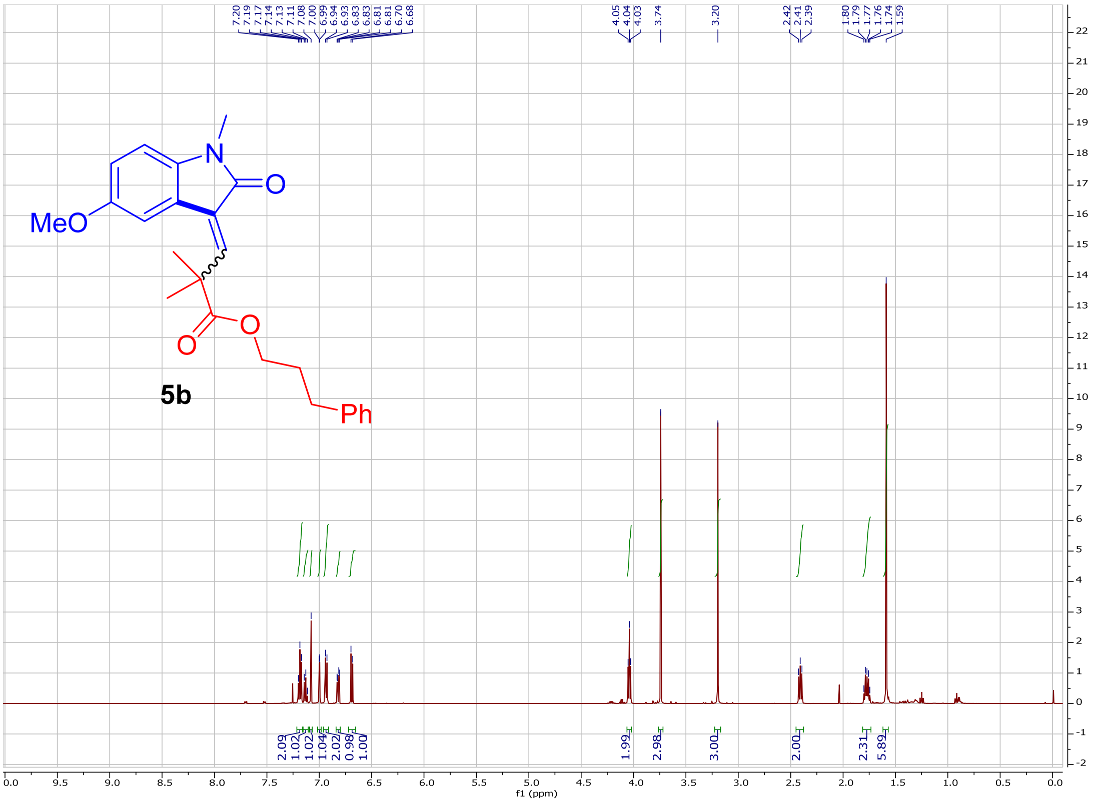

^{13}C NMR($CDCl_3$)

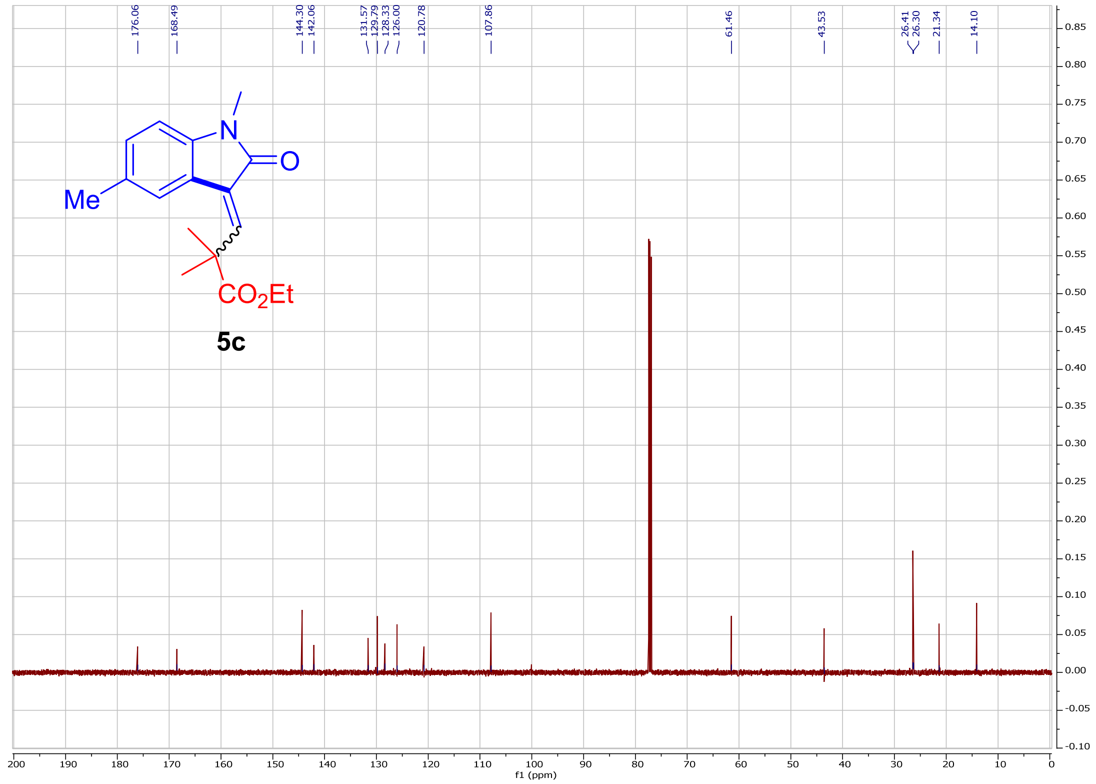

^{1}H NMR($CDCl_3$)

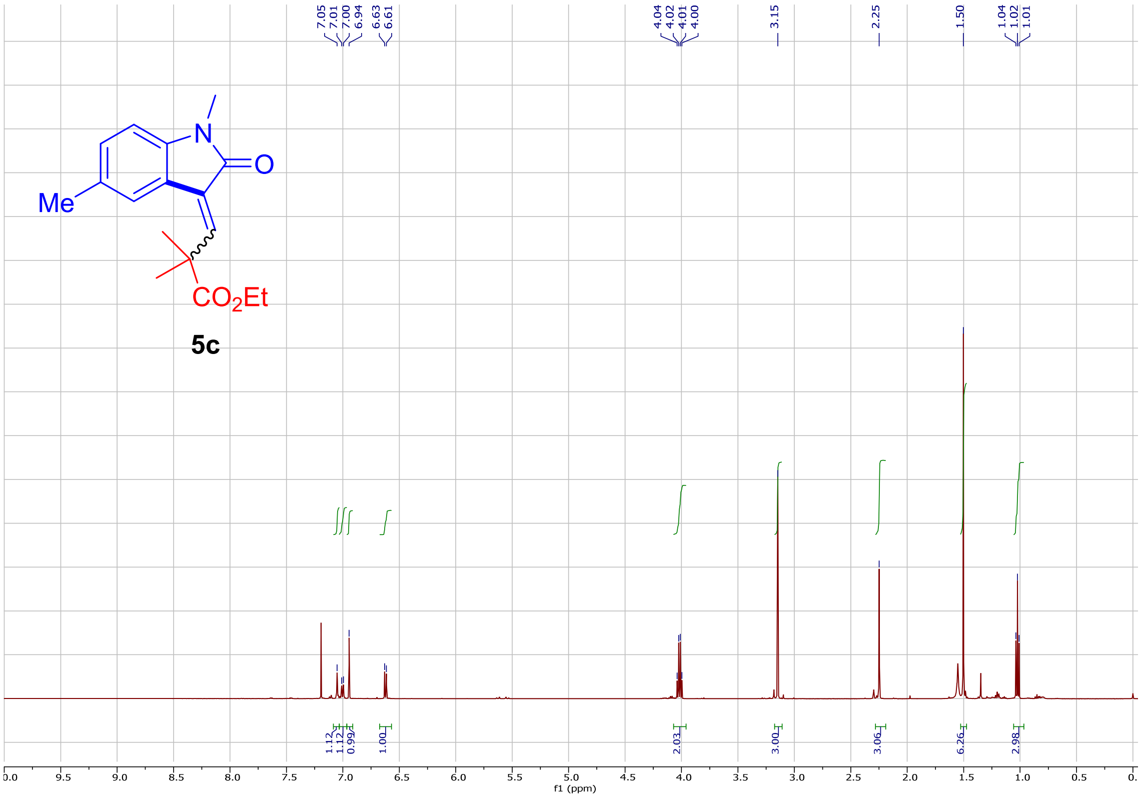

NOESY

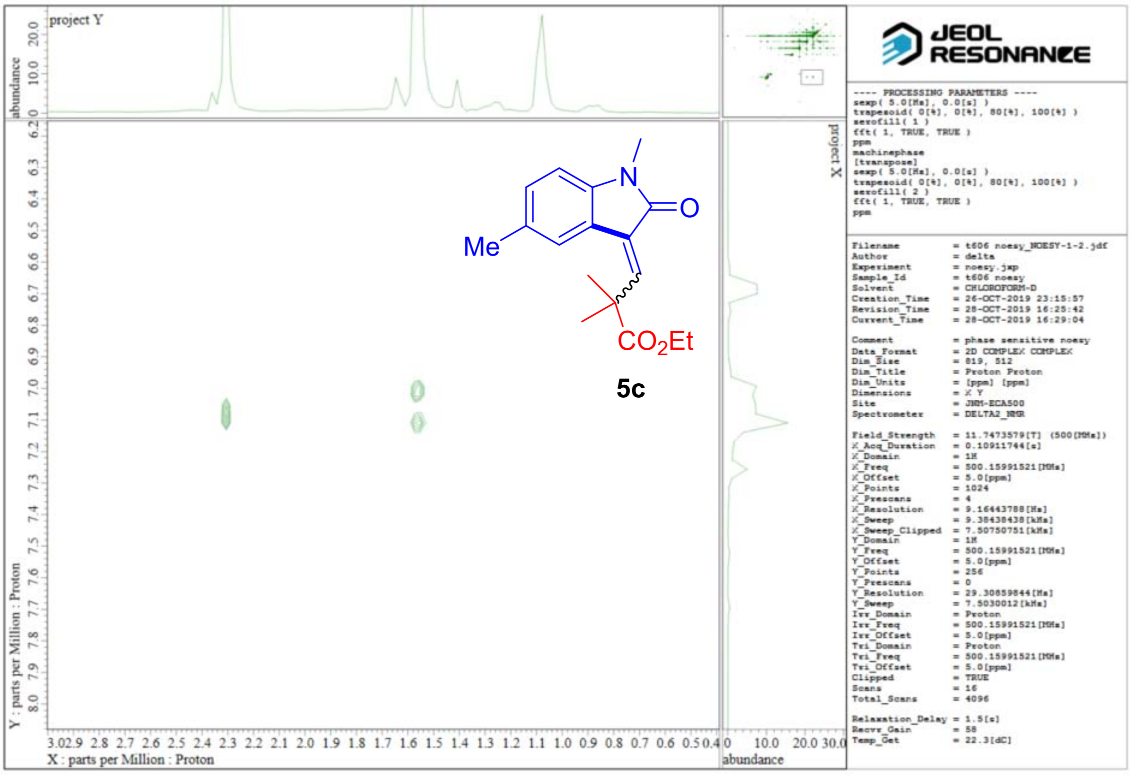

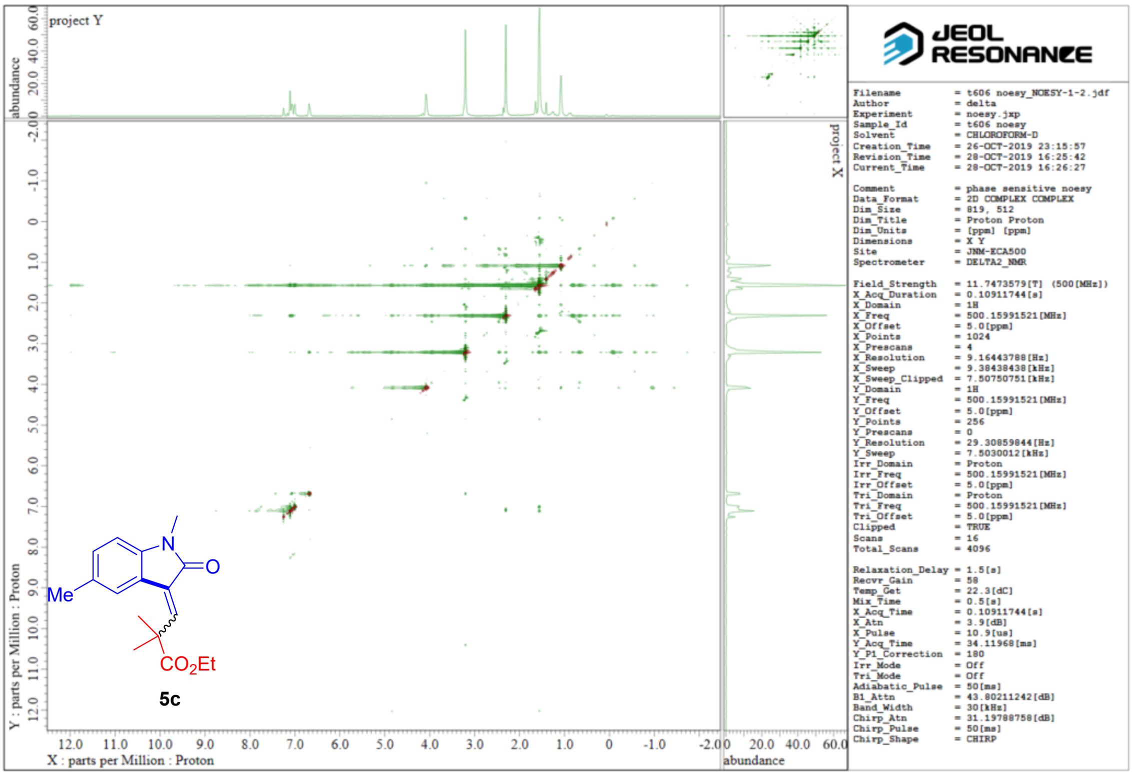

^{13}C NMR(CDCl3)

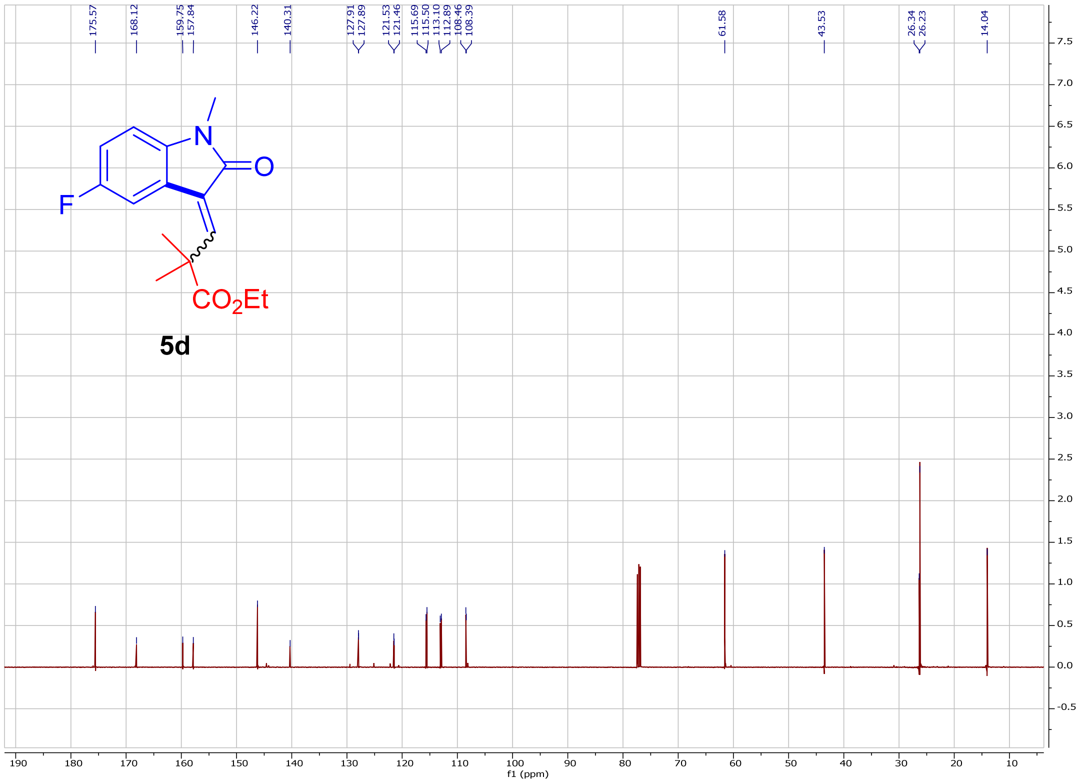

^{1}H NMR(CDCl3)

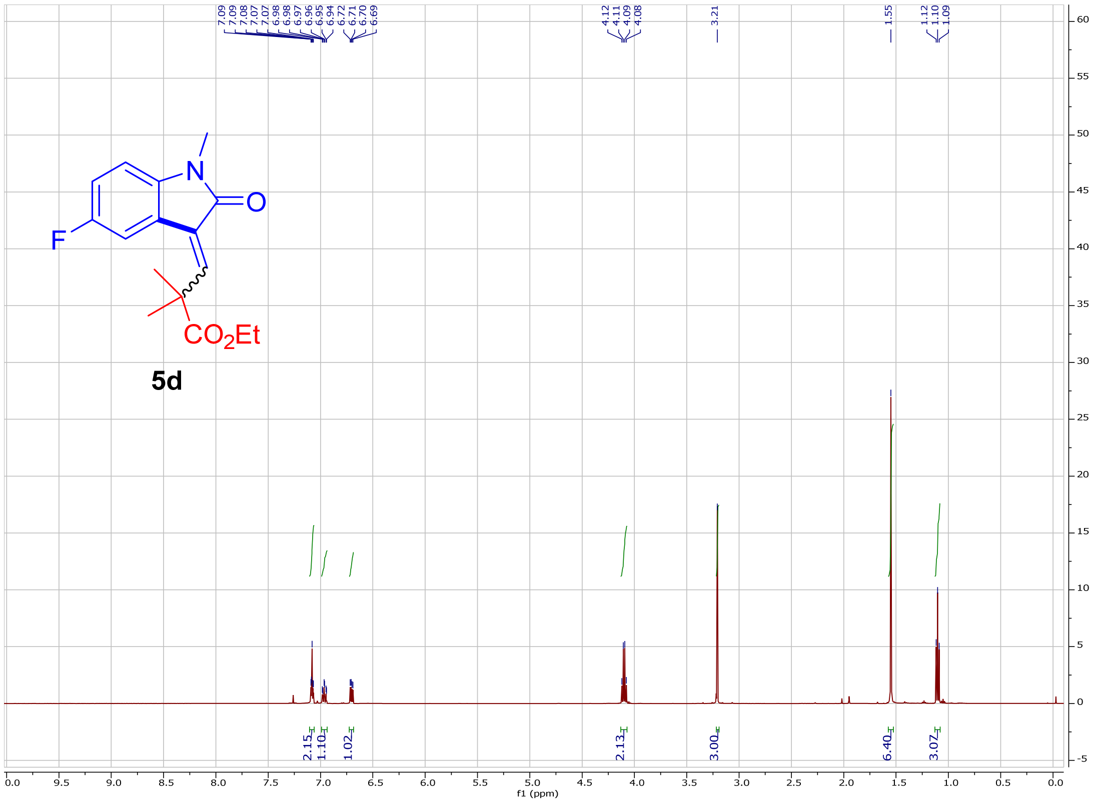

^{13}C NMR($CDCl_3$)

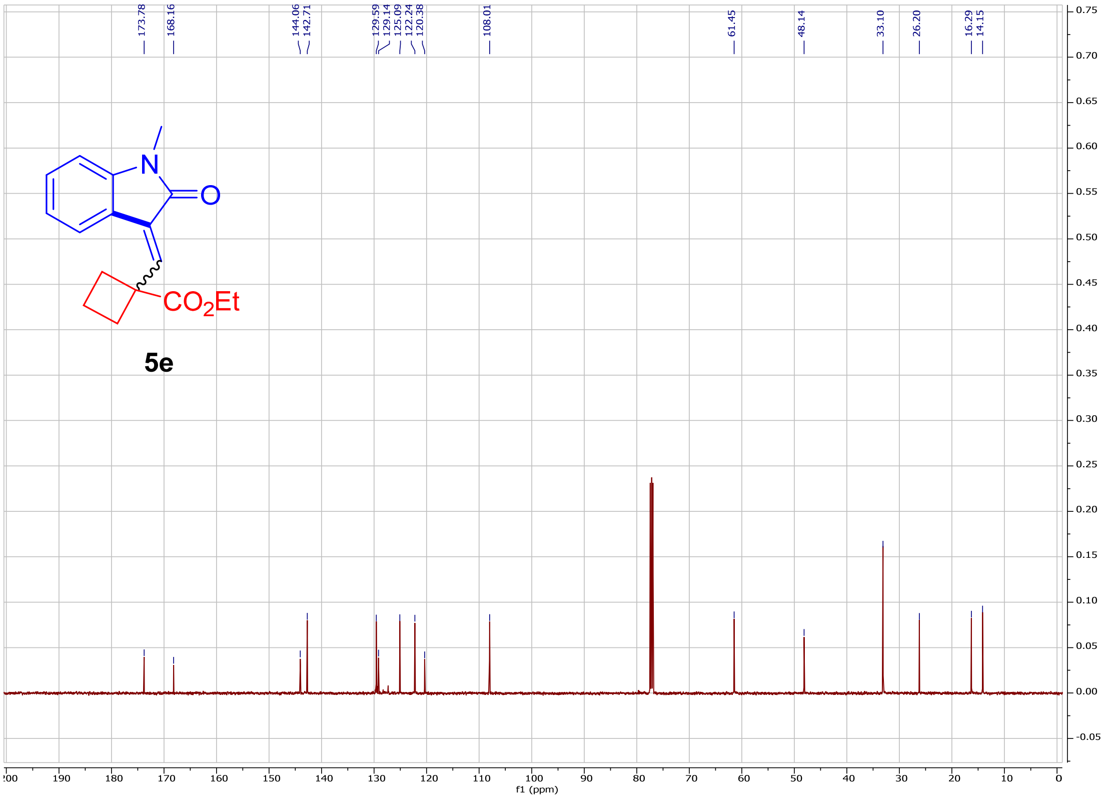

^{1}H NMR($CDCl_3$)

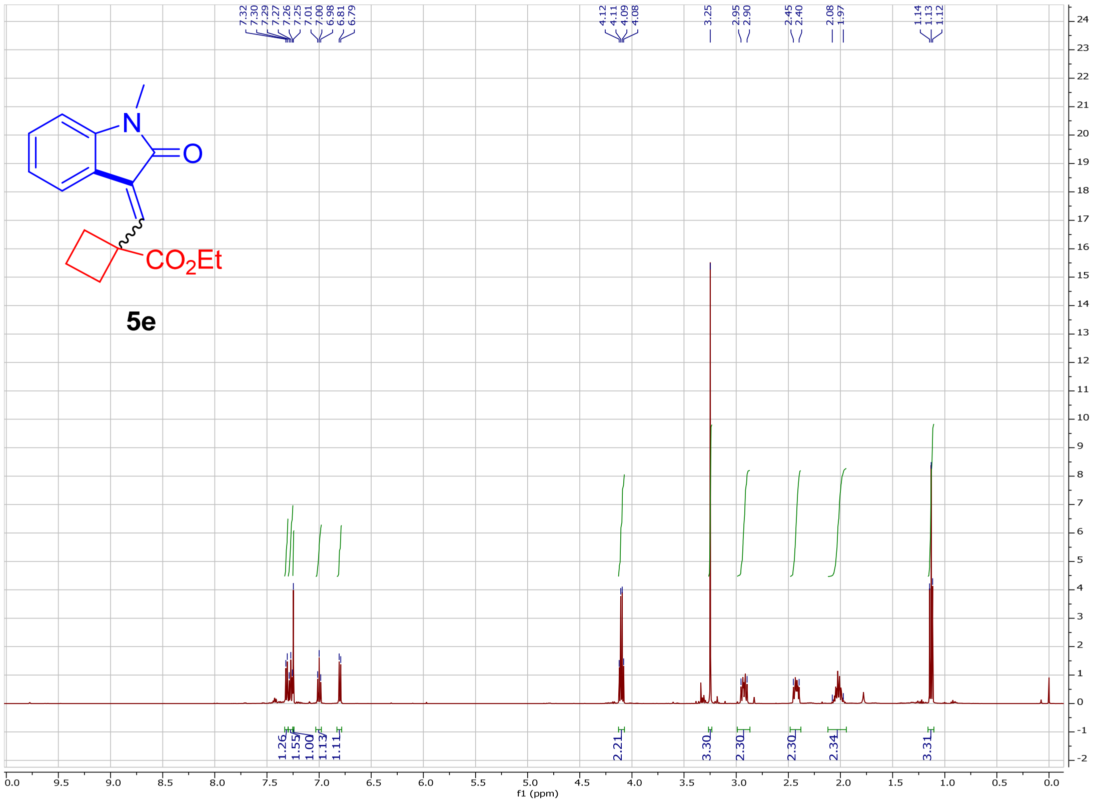

NOESY

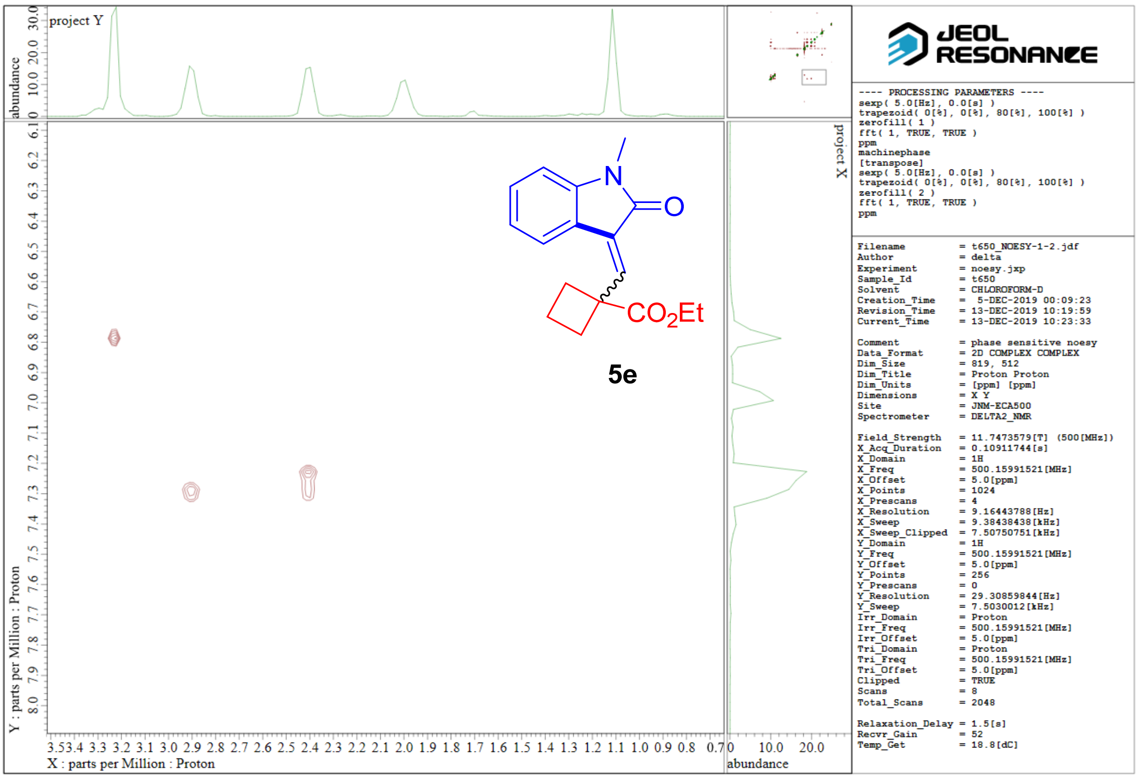
project Y
abundance
project X
5e
N
O
CO_2Et
JEOL RESONANCE
---- PROCESSING PARAMETERS ----
sexp(5.0[Hz], 0.0[s])
trapezoid(0[%], 0[%], 80[%], 100[%])
zerofill(1)
fft(1, TRUE, TRUE)
ppm
machinephase
[transpose]
sexp(5.0[Hz], 0.0[s])
trapezoid(0[%], 0[%], 80[%], 100[%])
zerofill(2)
fft(1, TRUE, TRUE)
ppm
Filename = t650_NOESY-1-2.jdf
Author = delta
Experiment = noesy.jxp
Sample_Id = t650
Solvent = CHLOROFORM-D
Creation_Time = 5-DEC-2019 00:09:23
Revision_Time = 13-DEC-2019 10:19:59
Current_Time = 13-DEC-2019 10:23:33
Comment = phase sensitive noesy
Data_Format = 2D COMPLEX COMPLEX
Dim_Size = 819, 512
Dim_Title = Proton Proton
Dim_Units = [ppm] [ppm]
Dimensions = X Y
Site = JNM-ECA500
Spectrometer = DELTA2_NMR
Field_Strength = 11.7473579[T] (500[MHz])
X_Acq_Duration = 0.10911744[s]
X_Domain = 1H
X_Freq = 500.15991521[MHz]
X_Offset = 5.0[ppm]
X_Points = 1024
X_Prescans = 4
X_Resolution = 9.16443788[Hz]
X_Sweep = 9.38438438[kHz]
X_Sweep_Clipped = 7.50750751[kHz]
Y_Domain = 1H
Y_Freq = 500.15991521[MHz]
Y_Offset = 5.0[ppm]
Y_Points = 256
Y_Prescans = 0
Y_Resolution = 29.30859844[Hz]
Y_Sweep = 7.5030012[kHz]
Irr_Domain = Proton
Irr_Freq = 500.15991521[MHz]
Irr_Offset = 5.0[ppm]
Tri_Domain = Proton
Tri_Freq = 500.15991521[MHz]
Tri_Offset = 5.0[ppm]
Clipped = TRUE
Scans = 8
Total_Scans = 2048
Relaxation_Delay = 1.5[s]
Recvr_Gain = 52
Temp_Get = 18.8[dC]
Y : parts per Million : Proton
X : parts per Million : Proton

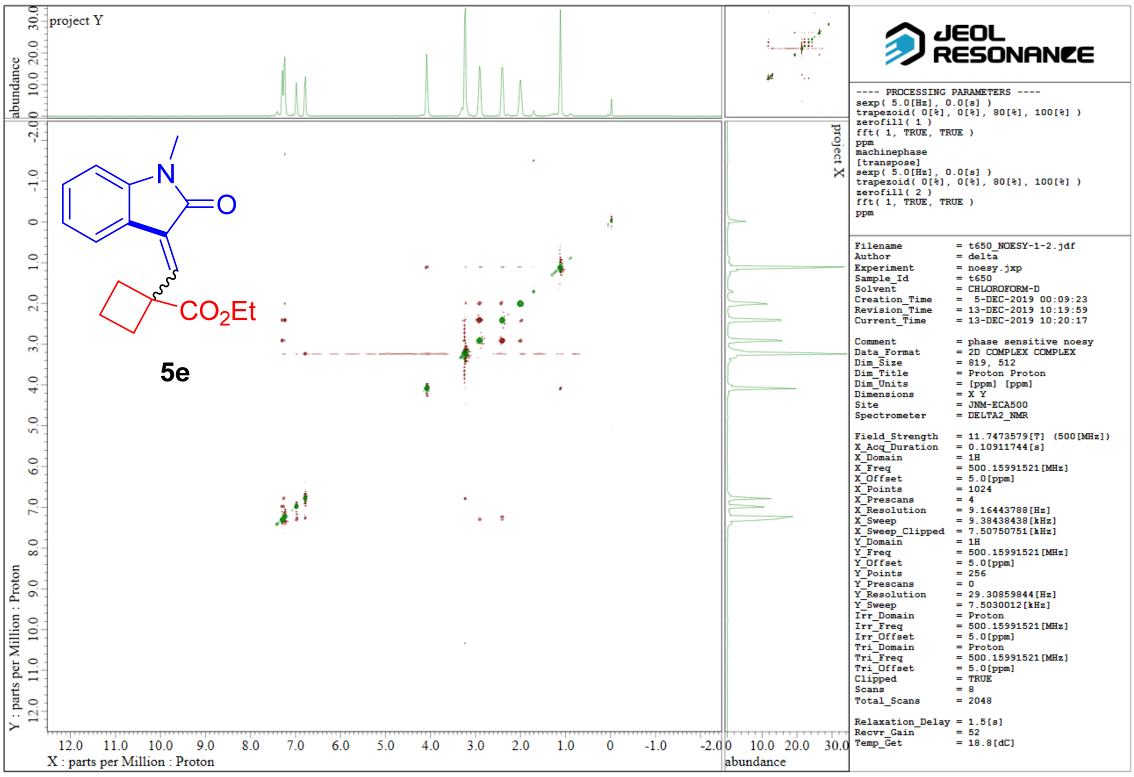
project Y
abundance
project X
5e
N
O
CO_2Et
JEOL RESONANCE
---- PROCESSING PARAMETERS ----
sexp(5.0[Hz], 0.0[s])
trapezoid(0[%], 0[%], 80[%], 100[%])
zerofill(1)
fft(1, TRUE, TRUE)
ppm
machinephase
[transpose]
sexp(5.0[Hz], 0.0[s])
trapezoid(0[%], 0[%], 80[%], 100[%])
zerofill(2)
fft(1, TRUE, TRUE)
ppm
Filename = t650_NOESY-1-2.jdf
Author = delta
Experiment = noesy.jxp
Sample_Id = t650
Solvent = CHLOROFORM-D
Creation_Time = 5-DEC-2019 00:09:23
Revision_Time = 13-DEC-2019 10:19:59
Current_Time = 13-DEC-2019 10:20:17
Comment = phase sensitive noesy
Data_Format = 2D COMPLEX COMPLEX
Dim_Size = 819, 512
Dim_Title = Proton Proton
Dim_Units = [ppm] [ppm]
Dimensions = X Y
Site = JNM-ECA500
Spectrometer = DELTA2_NMR
Field_Strength = 11.7473579[T] (500[MHz])
X_Acq_Duration = 0.10911744[s]
X_Domain = 1H
X_Freq = 500.15991521[MHz]
X_Offset = 5.0[ppm]
X_Points = 1024
X_Prescans = 4
X_Resolution = 9.16443788[Hz]
X_Sweep = 9.38438438[kHz]
X_Sweep_Clipped = 7.50750751[kHz]
Y_Domain = 1H
Y_Freq = 500.15991521[MHz]
Y_Offset = 5.0[ppm]
Y_Points = 256
Y_Prescans = 0
Y_Resolution = 29.30859844[Hz]
Y_Sweep = 7.5030012[kHz]
Irr_Domain = Proton
Irr_Freq = 500.15991521[MHz]
Irr_Offset = 5.0[ppm]
Tri_Domain = Proton
Tri_Freq = 500.15991521[MHz]
Tri_Offset = 5.0[ppm]
Clipped = TRUE
Scans = 8
Total_Scans = 2048
Relaxation_Delay = 1.5[s]
Recvr_Gain = 52
Temp_Get = 18.8[dC]
Y : parts per Million : Proton
X : parts per Million : Proton

^{13}C NMR(CDCl$_3$)

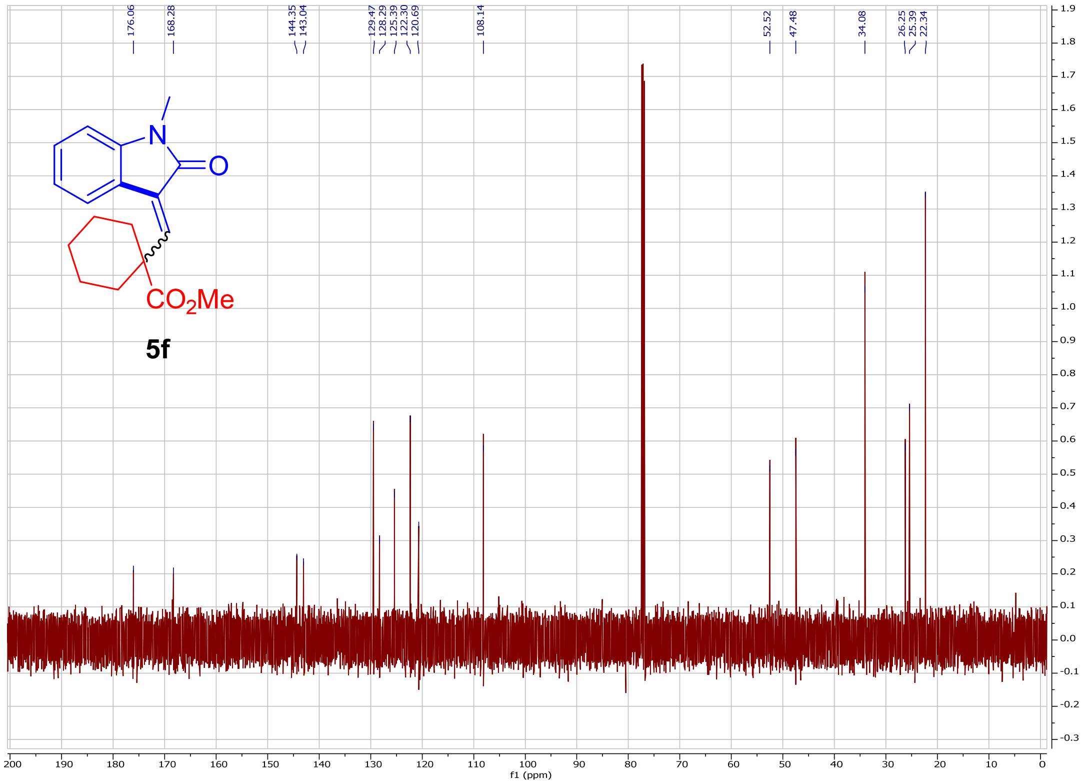

^{1}H NMR(CDCl$_3$)

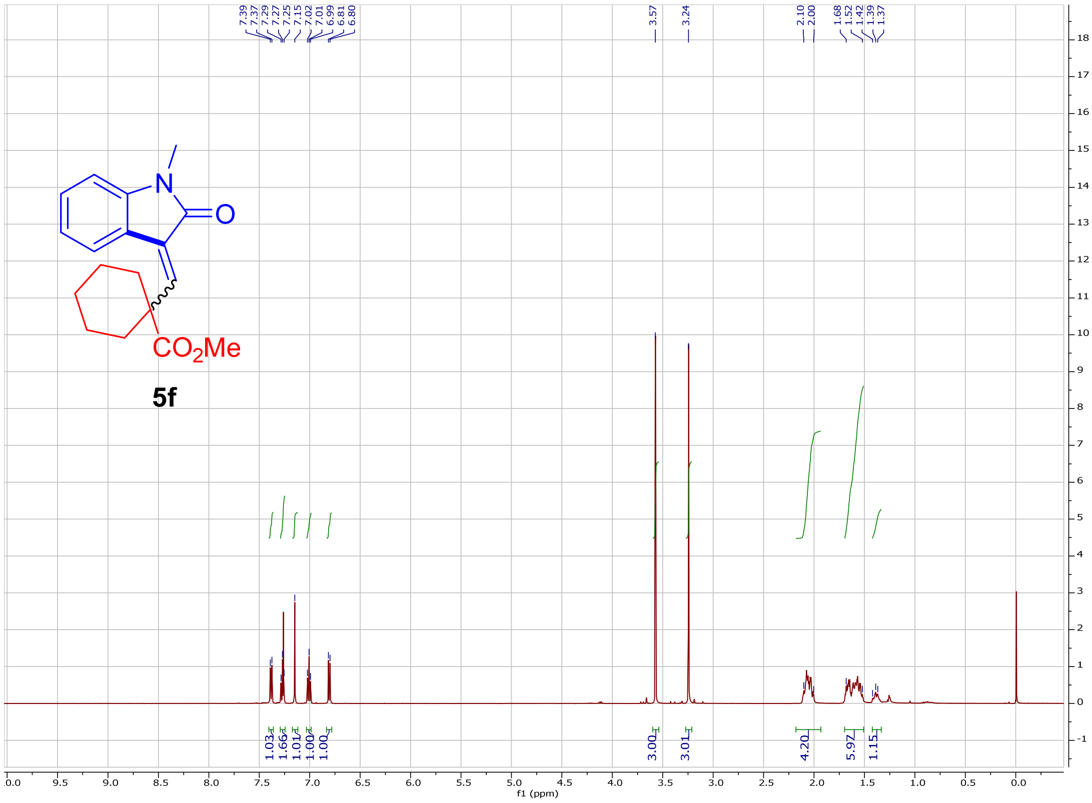

NOESY

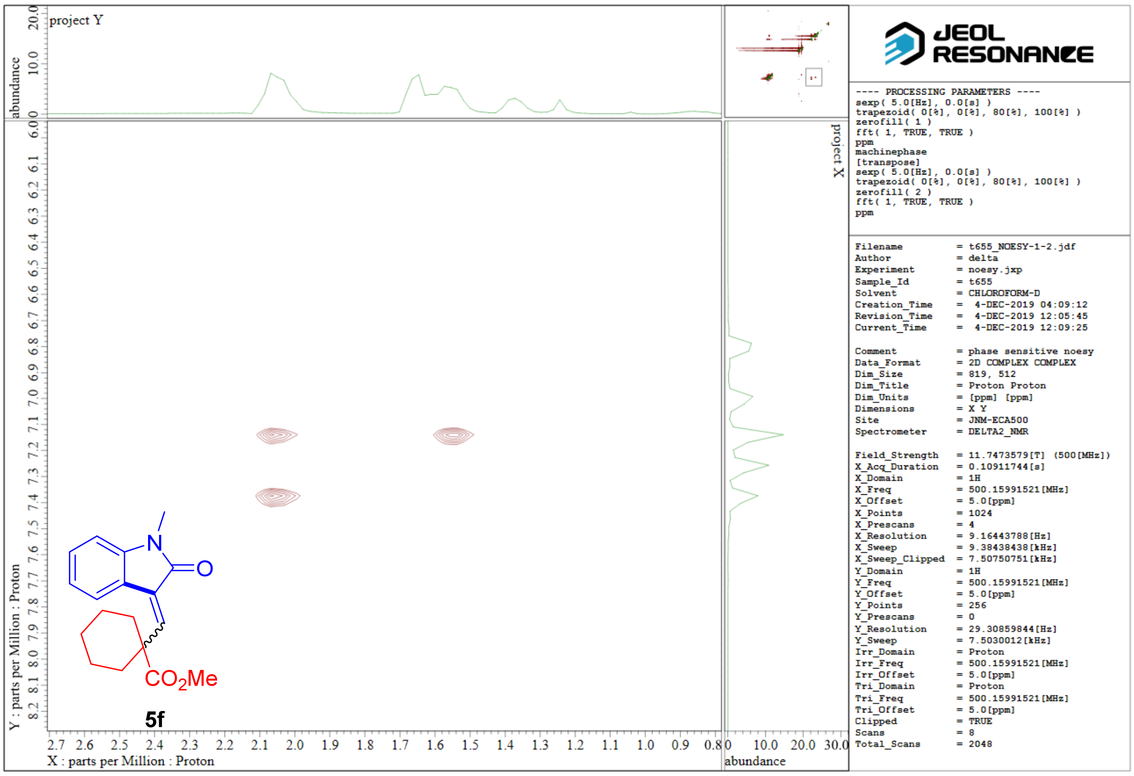
project Y
abundance
project X
JEOL RESONANCE
---- PROCESSING PARAMETERS ----
Filename = t655_NOESY-1-2.jdf
Author = delta
Experiment = noesy.jxp
Sample_Id = t655
Solvent = CHLOROFORM-D
Comment = phase sensitive noesy
Site = JNM-ECA500
Spectrometer = DELTA2_NMR
Field_Strength = 11.7473579[T] (500[MHz])
Scans = 8
Total_Scans = 2048
Y : parts per Million : Proton
X : parts per Million : Proton
CO2Me
5f

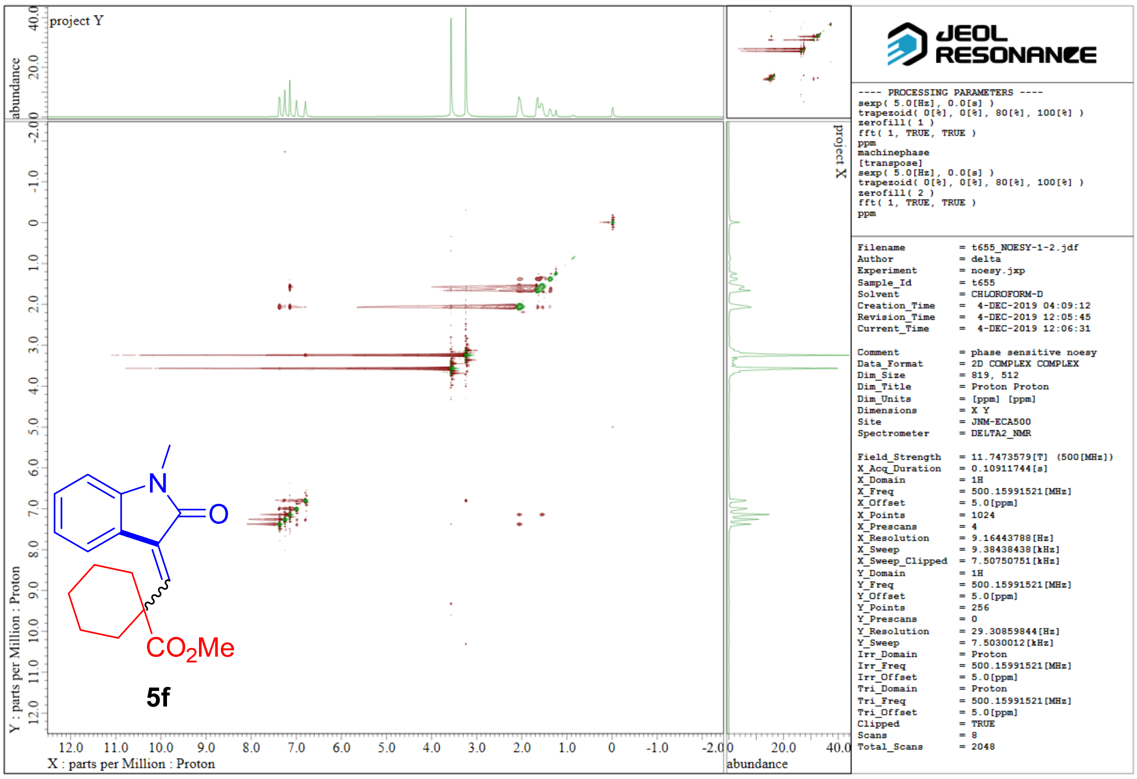
project Y
abundance
project X
JEOL RESONANCE
---- PROCESSING PARAMETERS ----
Filename = t655_NOESY-1-2.jdf
Author = delta
Experiment = noesy.jxp
Sample_Id = t655
Solvent = CHLOROFORM-D
Comment = phase sensitive noesy
Site = JNM-ECA500
Spectrometer = DELTA2_NMR
Field_Strength = 11.7473579[T] (500[MHz])
Scans = 8
Total_Scans = 2048
Y : parts per Million : Proton
X : parts per Million : Proton
CO2Me
5f

^{13}C NMR($CDCl_3$)

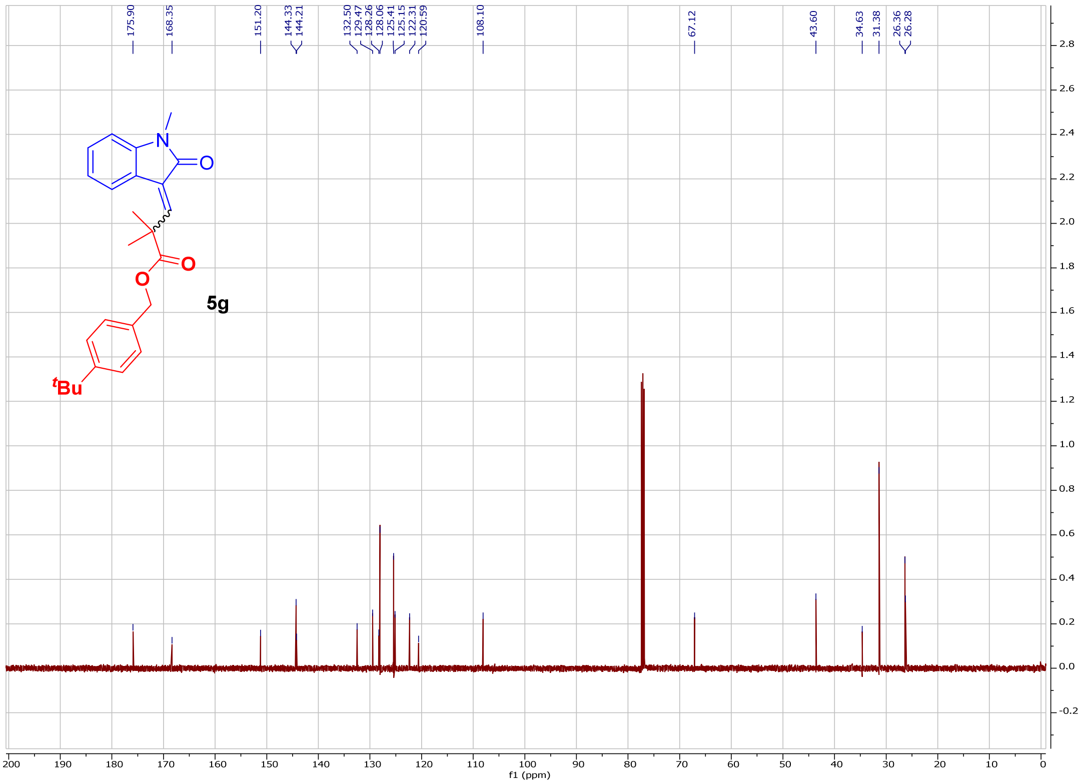

^{1}H NMR($CDCl_3$)

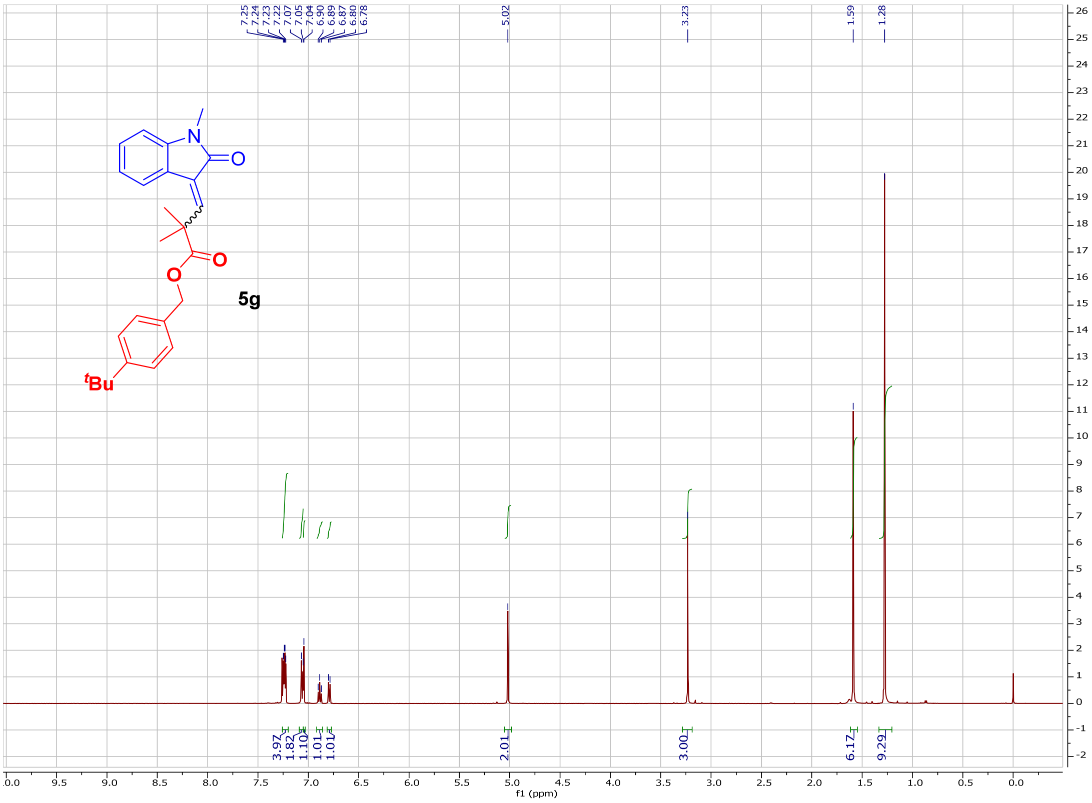

^{13}C NMR($CDCl_3$)

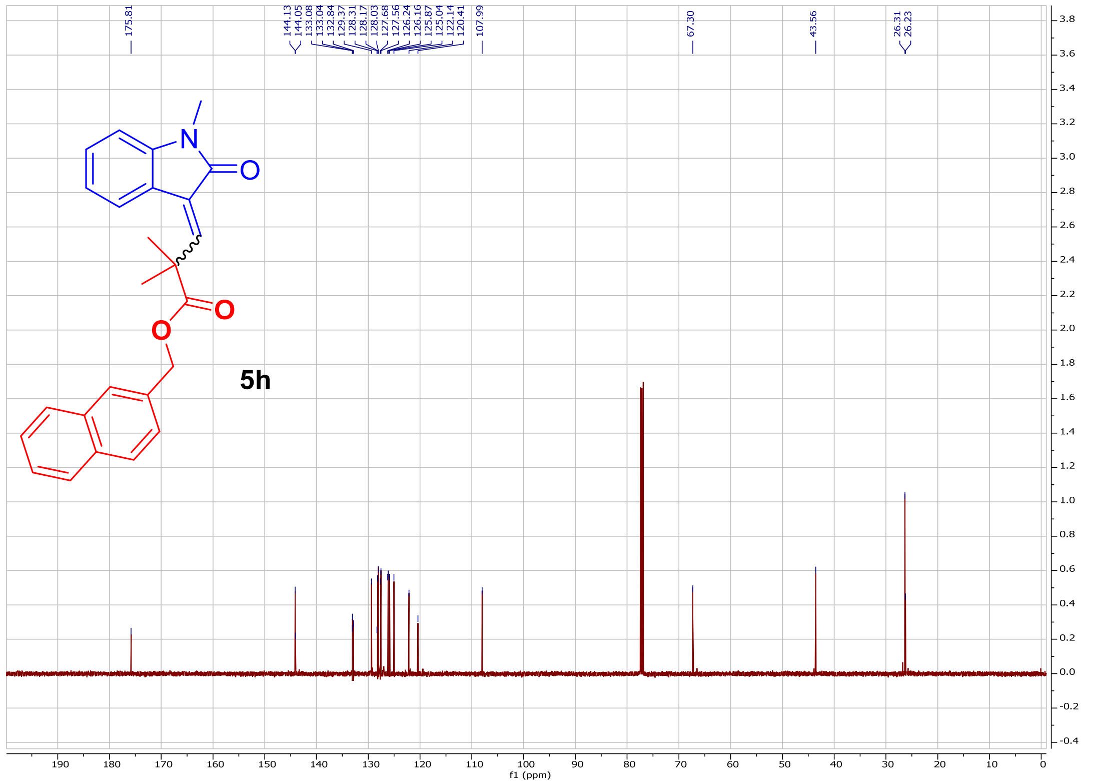

^{1}H NMR($CDCl_3$)

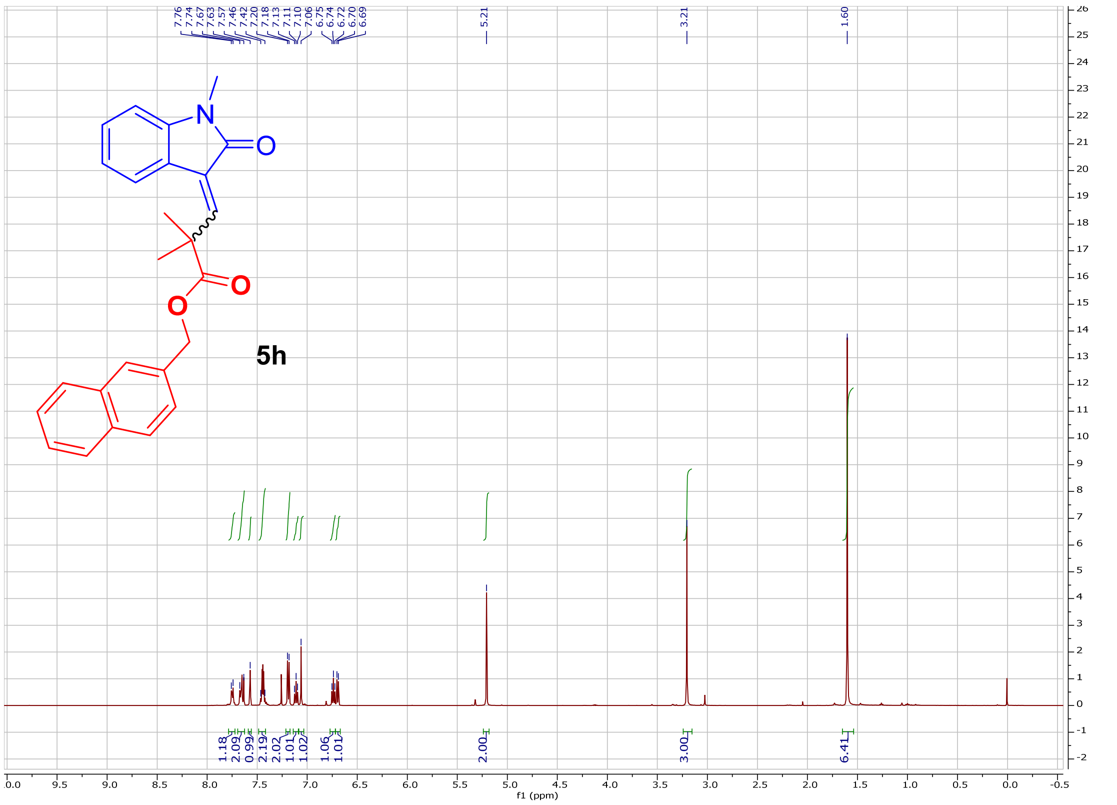

^{13}C NMR($CDCl_3$)

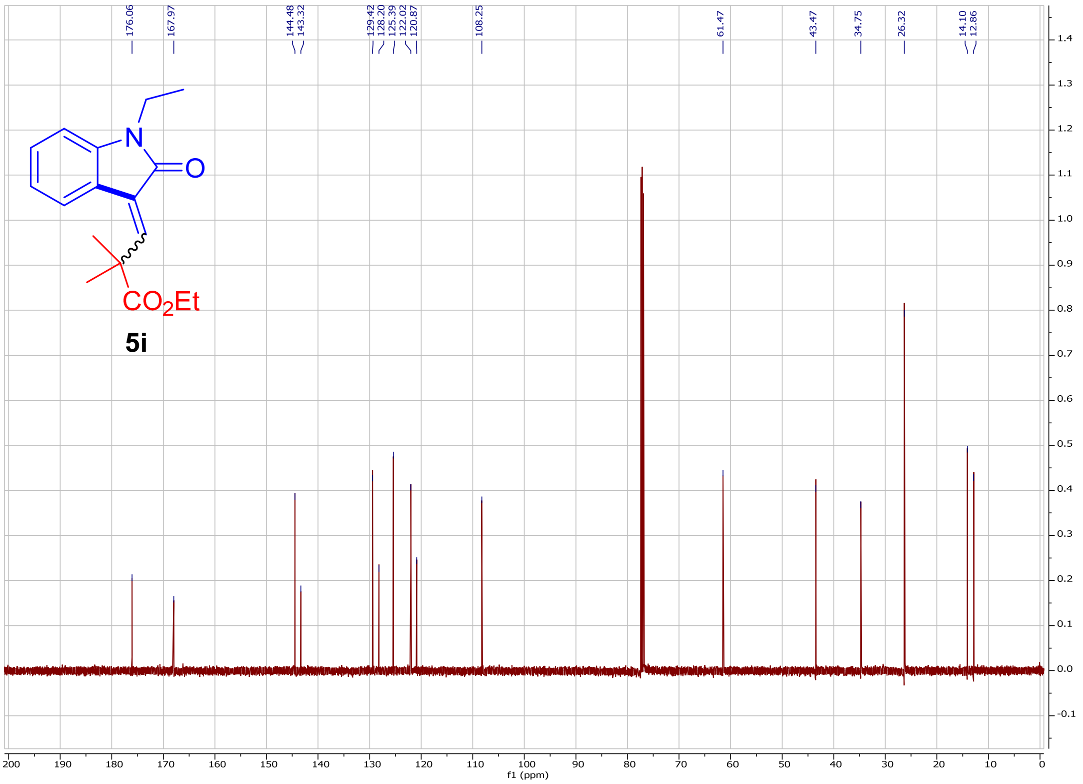

^{1}H NMR($CDCl_3$)

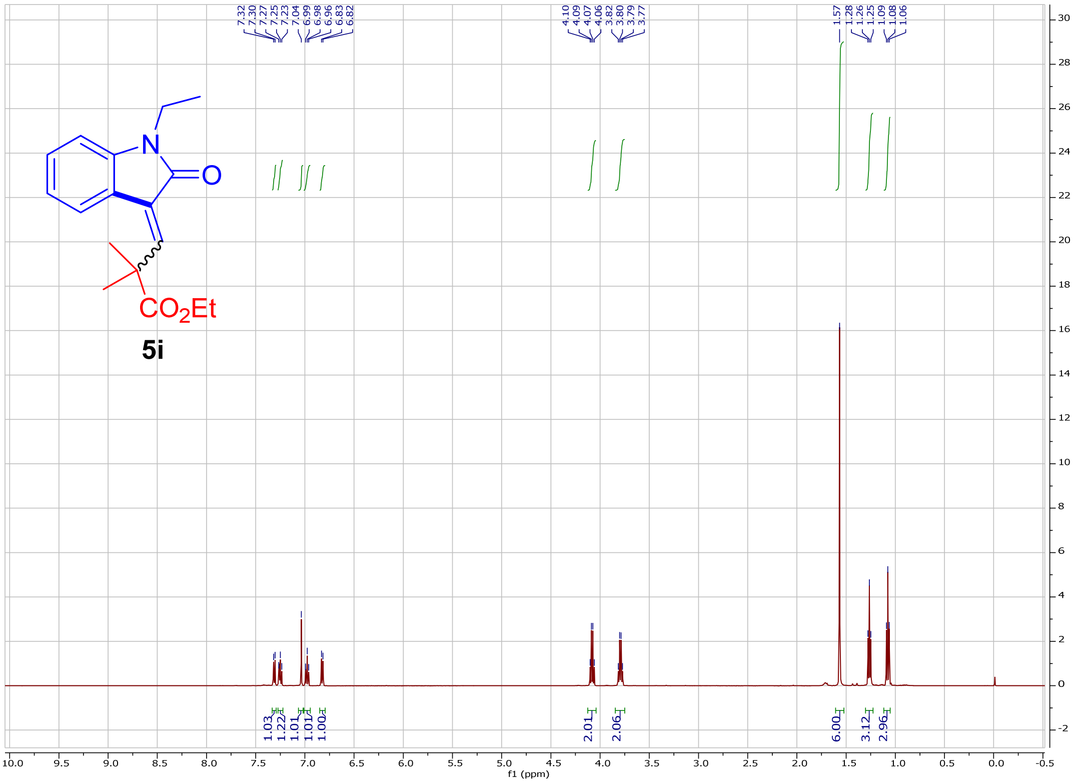

NOESY

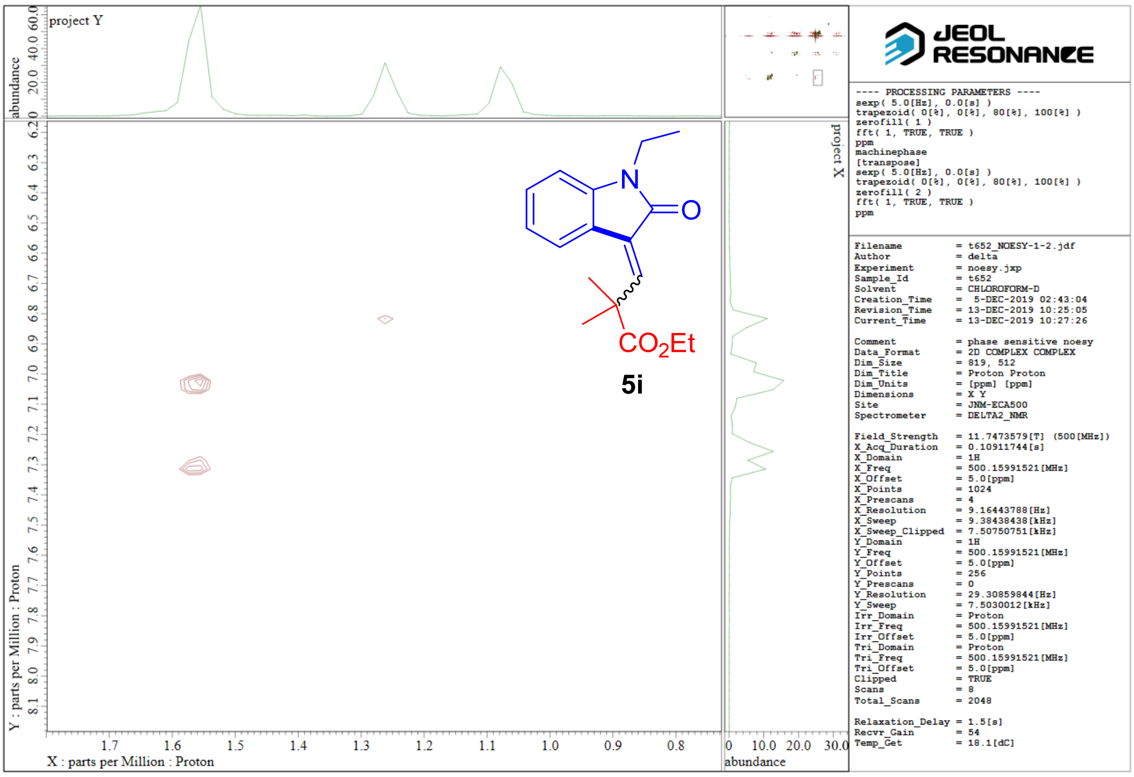

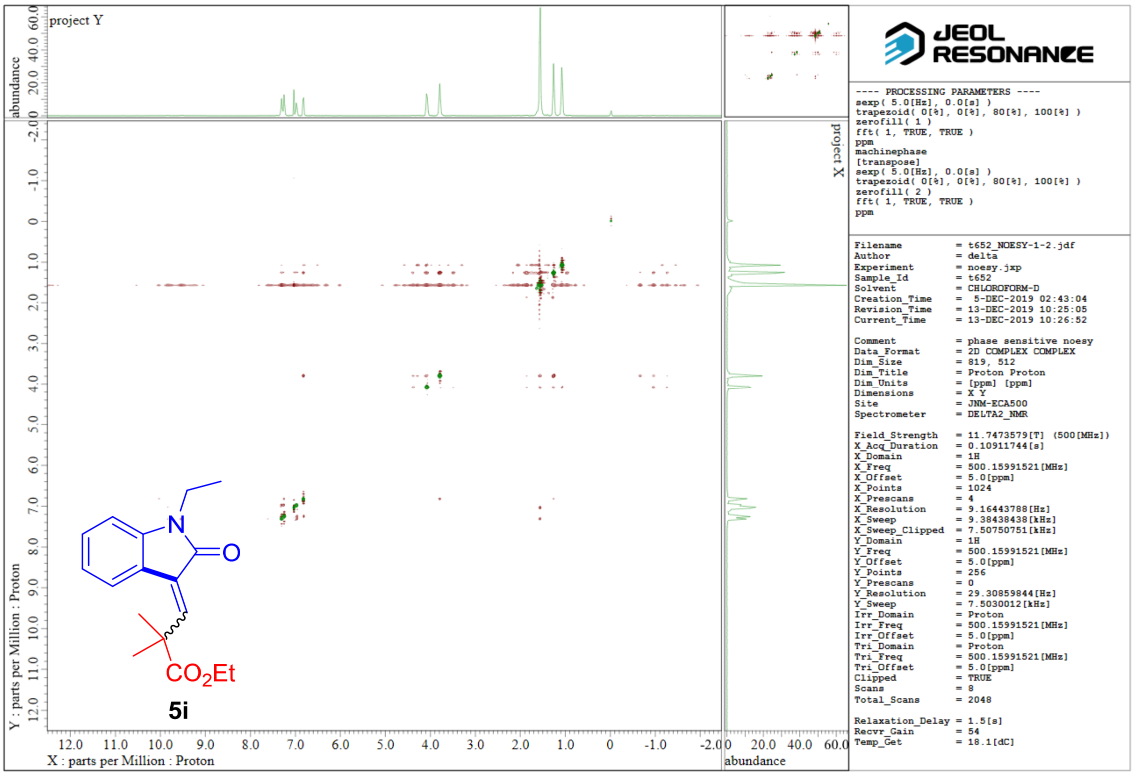

Supplement: File 1 — Experimental procedures, compound characterization data, and NMR spectra. [file Beilstein_J_Org_Chem-16-502-s001.pdf]
